# GPR55 in the tumor microenvironment of pancreatic cancer controls tumorigenesis

**DOI:** 10.3389/fimmu.2024.1513547

**Published:** 2025-01-16

**Authors:** Dušica Ristić, Thomas Bärnthaler, Eva Gruden, Melanie Kienzl, Laura Danner, Karolina Herceg, Arailym Sarsembayeva, Julia Kargl, Rudolf Schicho

**Affiliations:** Division of Pharmacology, Otto Loewi Research Center, Medical University of Graz, Graz, Austria

**Keywords:** KPCY model, pancreatic cancer, GPR55, checkpoint inhibitors, anti-PD-1 antibody, tumor microenvironment, CXCR3/CXCL9 axis

## Abstract

**Background:**

The G protein-coupled receptor 55 (GPR55) is part of an expanded endocannabinoid system (ECS), and plays a pro-tumorigenic role in different cancer models, including pancreatic cancer. Next to cancer cells, various cells of the immune tumor microenvironment (TME) express receptors of the ECS that critically determine tumor growth. The role of GPR55 in cancer cells has been widely described, but its role in the immune TME is not well understood.

**Methods:**

We intended to uncover the role of GPR55 in tumor immunity in a model of pancreatic ductal adenocarcinoma (PDAC). To this end, a KPCY tumor cell line or a GPR55-overexpressing KPCY cell line (KPCY55) from murine PDAC were subcutaneously injected into wildtype (WT) and GPR55 knockout (KO) mice, and immune cell populations were evaluated by flow cytometry.

**Results:**

Deficiency of GPR55 in the TME led to reduced tumor weight and volume, and altered the immune cell composition of tumors, favoring an anti-tumorigenic environment by increasing the number of CD3^+^ T cells, particularly CD8^+^ T cells, and the expression of PDL1 on macrophages. RNA-seq pathway analysis revealed higher T cell activity in KPCY55 tumors of GPR55 KO vs. WT mice. In addition, tumors from GPR55 KO mice displayed increased levels of T cell chemokines Cxcl9 and Cxcl10. Migration of T cells from GPR55 KO mice towards CXCL9 was increased in comparison to T cells from WT mice, suggesting that a CXCR3/CXCL9 axis was involved in T cell influx into tumors of GPR55 KO mice. Notably, anti-PD-1 immunotherapy increased tumor burden in WT mice, while this effect was absent in the GPR55 KO mice.

**Conclusion:**

Our study indicates that GPR55 in TME cells may drive tumor growth by suppressing T cell functions, such as migration, in a model of PDAC, making it an interesting target for immunotherapies.

## 1 Introduction

The tumor microenvironment (TME) is comprised of a variety of cell types, such as immune, stromal, and endothelial cells, which play a crucial role in cancer progression (1). For instance, patients with abundant presence of cytotoxic T and natural killer (NK) cells in the TME have a better prognosis across multiple cancer types (2, 3). However, this favorable response may be hampered by other, namely immunosuppressive cell types present in the TME, like regulatory T cells (Tregs) and myeloid-derived suppressor cells (MDSCs) (4). Pancreatic ductal adenocarcinoma (PDAC) has a highly immune-suppressive TME, in part because the majority of infiltrating immune cells are myeloid in origin (5). Moreover, only a fraction of pancreatic cancer patients has infiltrating cytotoxic T cells, which are known to be correlated with improved survival (6). Lately, the endocannabinoid system (ECS) has attracted attention regarding its effects on the behavior of immune cells during inflammation and cancer (7, 8). Main members of the ECS are the cannabinoid receptors 1 and 2 (CB_1_ and CB_2_), their endogenous ligands (endocannabinoids), and enzymes for endocannabinoid metabolism, like monoacylglycerol lipase (MGL), the 2-arachidonoylglycerol (2-AG)-degrading enzyme (7). Other receptors show responsiveness to endocannabinoids, such as the G protein-coupled receptor 55 (GPR55), which is considered part of an ‘expanded’ ECS, or the ‘endocannabinoidome’ (9–11). Various immune cells in the TME possess cannabinoid receptors (7), which can be influenced by components of the ECS (9). We previously showed prominent expression of CB_2_ and MGL in TME immune cells of non-small cell lung cancer (NSCLC) models and human NSCLC tissue (12, 13). Like CB_2_ (14), GPR55 is present in immune cells, e.g., B cells (15), T cells (16) and neutrophils (17), and also in cancer cells of various origin, for instance, in colon and pancreatic cancer cells (18–20). In many types of cancer that have been studied, GPR55 primarily plays a pro-tumorigenic role (18, 20–25). As to pancreatic cancer, genetic ablation of GPR55 in a PDAC model clearly improved disease outcome (20).

Since involvement of GPR55 in cancerogenesis was mostly researched in the context of its role in cancer cells (18, 19, 26), we focused on the immune TME in this study using GPR55 KO mice, and explored whether the knockout of GPR55 could have an influence on the immune cell landscape and the tumor progression in a PDAC mouse model (27). To this end, we used KPCY tumor cells (from mouse PDAC), and - since GPR55 is highly expressed in human pancreatic cancer cells (20) and higher stage pancreatic intraepithelial neoplasia (28) - KPCY tumor cells that overexpressed GPR55 (termed KPCY55). KPCY and KPCY55 cell lines were subcutaneously (s.c.) injected into the flanks of immunocompetent wildtype (WT) or GPR55 knockout (KO) mice. By using GPR55 KO mice in this model, we created a situation where GPR55 was present in cancer cells, but not in cells of the TME. We can report that mice lacking GPR55 had smaller tumors and higher lymphoid cell infiltration than WT mice. The results may have importance for developing new immunotherapies against PDAC.

## 2 Materials and methods

### 2.1 Cancer cell lines

The KPCY cell lines were generated from late-stage primary pancreatic tumors from C57BL/6 KPC mice expressing a YFP lineage tag (KPCY) (Trp53^L/+^) (27). One T-cell high clone (2838c3) was purchased from Kerafast (Boston, MA, USA). The KPCY cells were maintained in DMEM with 10% FBS (Life Technologies) and 1% penicillin/streptomycin (P/S, PAA Laboratories) at 37°C and 5% CO_2_ in a humidified atmosphere.

A GPR55-overexpressing KPCY cell line (KPCY55) was generated in our lab from the original KPCY parental cell line using a lentivirus for transduction that carried a GPR55 Puro cassette (or a control cassette) for stable overexpression of GPR55 (VectorBuilder). Puromycin (0.5 µg/ml, Thermo Fisher, A1113803) was used for positive selection of clones, and overexpression was confirmed with RT-qPCR.

### 2.2 Mice used in the study

C57BL6/J mice were purchased from Charles River (Germany) and bred in house. GPR55 KO mice (B6;129S-Gpr55^tm1Lex^/Mmnc) were obtained through MMRRC (Mutant Mouse Regional Resource Center; USA). The strain was backcrossed with C57BL6/J mice for ten generations, and was also bred in house (18). Experimental procedures were approved by the Austrian Federal Ministry of Science, Research and Economy (protocol # 2022-0.748.851) and performed in strict accordance with international guidelines. All experimental procedures were performed on 7-12-week male mice.

### 2.3 PDAC mouse model

KPCY and KPCY55 cells were dissociated into single cells, washed with PBS twice and counted before s.c. injection. Cells (5×10^5^) were resuspended in 450 μL Dulbecco’s phosphate buffered saline (PBS, Gibco) and injected into the flanks of mice on day 0. Tumors were harvested at the experimental endpoint upon reaching the appropriate size, i.e., on day 21 for the KPCY tumors, and on day 28 or 29 for the KPCY55 tumors. Tumors were then subsequently weighed, measured with a digital caliper *ex vivo*, and submitted to analysis. Tumor volume was calculated based on the following formula: V = length x width x height x π/6 (29).

### 2.4 Anti-PD1 antibody treatment

KPCY55 tumor-bearing WT or GPR55 KO mice were injected i.p. with 200 µg rat monoclonal anti-mouse PD-1 antibody (clone 29F.1A12, BioXCell, Lebanon, NH) or rat IgG2a isotype control (clone 2A3, BioXCell, Lebanon, NH) 6 times (as shown in the treatment protocol scheme) over a period of two weeks.

### 2.5 Single-cell suspensions

Single cell suspensions of s.c. tumors were prepared as previously described (13). Briefly, tumors were cut into small pieces with scissors. The tumor pieces were afterwards digested with collagenase for 30 minutes at 37°C (CLS-1; 4.5 U/ml; Worthington) and DNase I (160 mU/ml; Worthington; LS002006), while rotating at 1000 rpm. After digestion, tissue was passed through a 40 μm-strainer, washed with staining buffer (SB; PBS + 2% FBS), followed by a wash in PBS only.

### 2.6 Flow cytometric phenotyping of immune cell populations

In order to exclude dead cells, single cell suspensions were incubated for 20 min in Fixable Viability Dye (FVD) (eFluor™ 780; eBioscience, #65-0865-18) in the dark at 4°C. The cells were initially incubated with 1 μg TruStain™ FcX (Biolegend, #101320), and afterwards stained in the dark at 4°C for 30 min with antibodies listed in **Supplementary Table S1**. From this point onwards, the staining protocol differed for intracellular immunofluorescent staining. For surface staining, samples were fixed with eBioscience™ IC Fixation Buffer (ThermoFisher Scientific, #00-8222-49) in the dark for 10 min at 4°C, resuspended in SB, and acquired within two weeks on a BD LSR Fortessa, running on FACSDiva software (BD Biosciences). Samples requiring intracellular staining were fixed with Fix/Perm solution from the BD Cytofix/Cytoperm™ Fixation/Permeabilization Kit (BD Biosciences, #554714) for 20 min in the dark at 4°C. Afterwards, they were washed twice with 1x Perm/Wash buffer and then incubated with the antibody in Perm/Wash buffer for 30 min at 4°C. The cells were washed twice in 1x Perm/Wash buffer and resuspended in SB prior to analysis. FlowJo software (v10.20, Treestar) was used for analysis and compensation. See **Supplementary Figures S1** and **S2** for gating strategies.

### 2.7 RNA extraction and RT-qPCR

RNA was extracted from tissue using Trizol (Life Technologies, #15596026). Samples were then treated either with a DNA-free™ DNA Removal Kit (Invitrogen, #10729525) or RNase-Free DNase set (Qiagen, #79254). Quality and concentration of RNA were determined using a NanoDrop ND-1000 spectrophotometer (Thermo Fisher Scientific). Reverse transcription of purified RNA (1 μg) was performed by High-Capacity cDNA Reverse Transcription Kit (Applied Biosystems, #4368814). Gene expression was assessed by reverse transcription-quantitative polymerase chain reaction (RT-qPCR) using SsoAdvanced Universal SYBR Green Supermix (Bio-Rad, #1725271). Primers were acquired from Eurofins (**Supplementary Table S2**). Relative gene expression was calculated using 2^-ΔCT^ methods (30). Hprt was used as a housekeeping gene.

### 2.8 Bulk RNA-seq: sample preparation and analysis

RNA was extracted from tissue using Trizol (Life Technologies, #15596026). Samples were afterwards treated with the DNA-free™ DNA Removal Kit (Invitrogen, #10729525), and the concentration and quality of RNA was measured using Agilent Bioanalyzer 2100. After poly-A enrichment and library prep, RNA-seq was performed on Illumina NovaSeq (6000 and the X Plus), with 20 million reads per sample (GENEWIZ, Azenta Life Sciences). The raw data were aligned to GRCm39 using STAR 2.7.10b, after running FastQC 0.11.9. Differential gene expression was assessed by RStudio 4.4.1 using edgeR package (version 4.2.0) (31). For pathway enrichment analysis, pathfindR was used (version 2.4.1) (32). Heatmaps were generated after normalization to transcripts per million and subsequent z-scaling. All RNA-seq data are deposited at GEO database, accession number GSE280636.

### 2.9 Isolation of splenic pan-T cells and migration assays

Pan-T cells were isolated from WT and GPR55 KO mouse spleens using EasySep™ Mouse T Cell Isolation Kit (Stemcell, #19851), according to the manufacturer’s protocol. T cell migration assay was performed using a 96-well MultiScreen plate (Millipore, MAMIC5S10) with a 5-μm pore-size polycarbonate filter. A series of dilutions of the chemokine (C-X-C motif) ligand 9 (CXCL9 Biolegend, #578204) in assay buffer containing Ca^2+^ and Mg^2+^ was pipetted into the bottom well, after which T cells were added to the top well in the same buffer (1x10^5^ per 75 μl). The cells were left to migrate in the plate for 90 min at 37°C. Migrated cells in the bottom wells were counted on a BD Accuri™ C6 Plus flow cytometer. Chemotactic index was defined as the number of cells migrating towards CXCL9 divided by the number of cells migrating towards negative control (17).

### 2.10 *In situ* hybridization and immunofluorescence

Tumors were fixed in acid-free phosphate-buffered 10% formaldehyde solution (Roti^®^-Histofix 10%, pH 7, Roth, P087.1) for 24-48 hrs at room temperature, and further processed for paraffin embedding, according to standard procedures. Tissue was cut in 5 μm-sections, baked at 60°C for 1 hr, dewaxed, and rehydrated. ISH was performed according to the manufacturer’s protocol, and as recently published by us (12). In brief, three ZZ probes for GPR55 (targeting bases 2-907 of NM_001033290.2) (RNAScope ™ RED kit; Advanced Cell Diagnostics [ACD], #322360) were used to detect the corresponding mRNAs in tumors. Sections with tumor tissue were treated with H_2_O_2_ for 10 min, which was followed by target retrieval, using the Brown FS3000 food steamer for 15 min. Each step was followed by washes in distilled water. The sections were digested with Protease IV at 40°C for 20 min, washed, incubated at 40°C for 2 hrs, and stained with FastRed. GPR55 KO and WT mice samples were put on one slide for comparison. GPR55 KO mice lacked expression of GPR55 outside of KPCY tumor cells (**Supplementary Figure S3**).

An antibody against GFP (1:500; Abcam #ab290) was used to label tumor cells (YFP-tagged). Antibodies against CD8^+^ (1:100; Abcam # ab203035), CD11b^+^ (1:100; Novus #NB11089474), F4/80^+^ (1:500, Cell Signaling #70076), and CD4^+^ (1:400, Abcam #ab183685) were used to stain cell types of the immune TME co-localizing with GPR55 mRNA. After ISH, tissue sections were first blocked with 5% goat serum (Sigma-Aldrich) in 0.1 M PBS containing 0.3% Triton X-100. Afterwards, primary antibodies were applied in 0.1 M PBS containing 0.3% Triton X-100 and 1% goat serum over night at 4°C. As second antibodies, Alexa Fluor^®^ 488 goat anti-rabbit (1:500; Jackson Immuno Research; #111-5491144) or Cy5-labeled anti-rabbit IgGs (1:500; Jackson Immuno Research; #711-175-152) were used. Sections were then mounted with Vector^®^ TrueVIEW^®^ Autofluorescence Quenching Kit containing DAPI (Vector Laboratories, #SP-8500-15), and images were taken by an Olympus IX73 fluorescence microscope (Olympus) connected with a Hamamatsu ORCA-ER digital camera (Hamamatsu Photonics K.K.). Images were processed with Olympus CellSense^®^ 1.17 imaging software (Olympus). Only contrast, brightness, and color balance of images were adjusted. ImageJ software and QuPath (version 0.4.3) were applied to quantify expression and colocalization with the ISH probe (29).

### 2.11 Statistical analysis

GraphPad Prism 10.0.3 (GraphPad^®^ Software) was used to perform statistical analyses. Data are presented as means ± standard deviation (SD), or as medians with 25^th^ and 75^th^ percentiles, and min-max values. Statistically significant differences between two experimental groups were determined by unpaired Student’s t-test, two-way ANOVA with Šídák’s multiple comparisons or Tukey’s *post-hoc* test, and by ordinary one-way ANOVA with Dunnett’s multiple comparisons test. P values <0.05 were considered significant and denoted with 1, 2, 3 or 4 asterisks when lower than 0.05, 0.01, 0.001, or 0.0001, respectively. For RNA-seq data analysis, edgeR (version 2.4.0) was used to obtain differential gene expression according to the respective vignette.

## 3 Results

### 3.1 GPR55 deficiency reduces KPCY-induced tumor weight and volume

**Figure 1:**
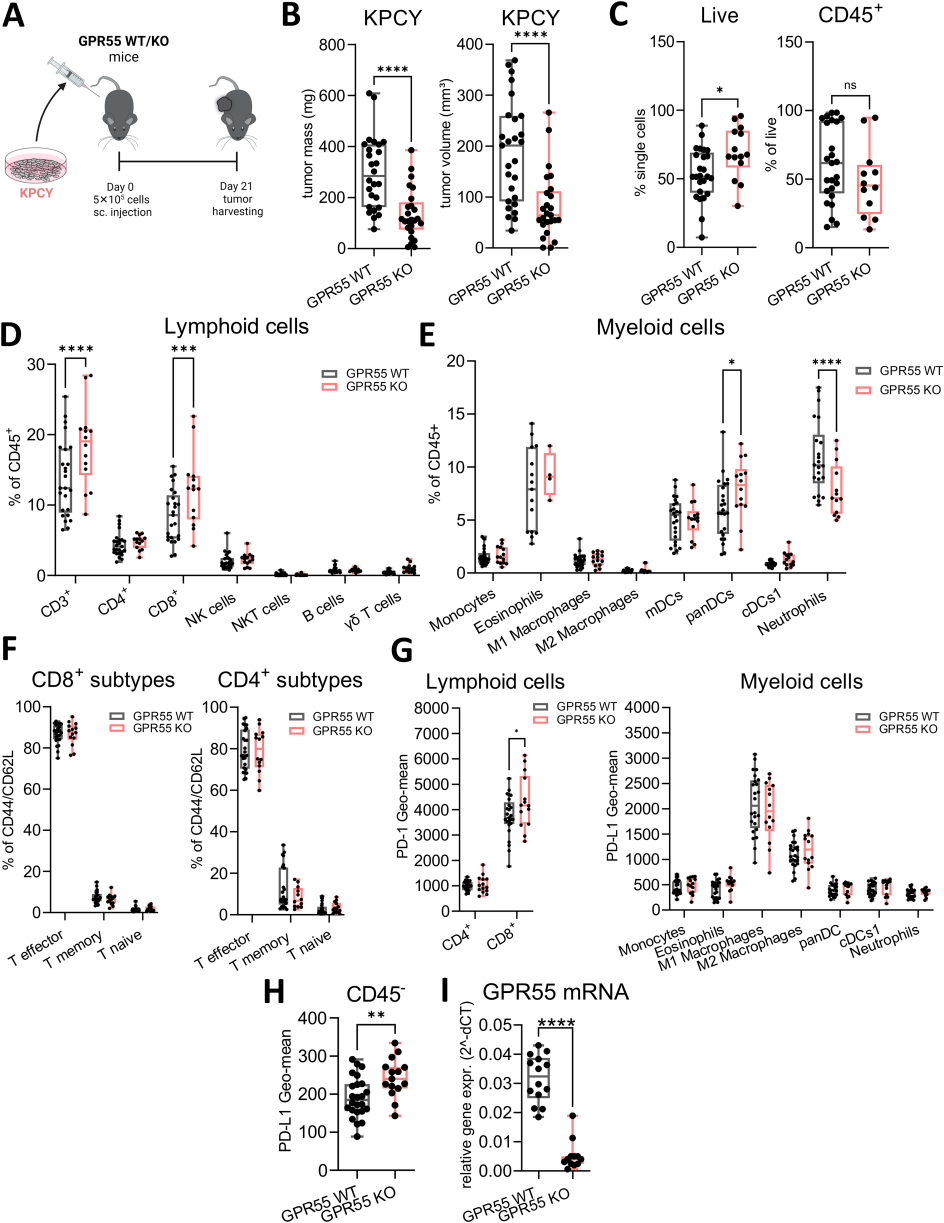
Deficiency of GPR55 reduces tumor weight and volume in KPCY-induced tumors. **(A)** GPR55 KO and WT mice were subcutaneously (s.c.) injected with 5x10^5^ KPCY cells on day 0. On day 21, tumors were measured *ex vivo* and processed for analysis. **(B)** Tumor mass and volume are expressed by medians, 25^th^ 75^th^ percentiles, and min-max values from three pooled independent experiments. n= 25-27. **(C–H)** Flow cytometric analysis of lymphoid and myeloid populations in single cell suspensions from KPCY tumors. Data indicate medians, 25^th^ and 75^th^ percentiles, and min-max values from three pooled independent experiments. n=14-25. **(I)** Relative gene expression of GPR55 mRNA in KPCY tumors. n=12-14. Statistical differences were evaluated by using unpaired Student’s t-test **(B, C, H, I)** or two-way ANOVA with Šídák’s multiple comparisons test **(D, E, G)**. *p<0.05; **p<0.01; ***p<0.001; ****p<0.0001. *WT*, wildtype; KO, knockout; *CD8^+^ T mem*, CD8^+^ T memory cells; *panDCs*, pan-dendritic cells; c*DCs1*, dendritic cell type 1; *mDCs*, monocyte-derived dendritic cells; *NK cells*, natural killer cells; ns, not significant; *γδ T cells*; *TME*, tumor microenvironment.

Tumors were induced by s.c. injection of KPCY cells and harvested after 21 days (**Figure 1A**). *Ex vivo* measurements revealed that the tumors of GPR55 KO mice were smaller in weight and volume than those of WT mice (**Figure 1B**). Although we measured more live cells in tumors from KO mice, percentages of CD45^+^ cells did not differ significantly between the two groups (**Figure 1C**). Percentages of CD3^+^ T cells, CD8^+^ T cells, and pan-dendritic cells were higher in tumors of GPR55 KO vs. WT mice (**Figures 1D, E**), while the number of neutrophils was lower (**Figure 1E**). We observed no changes in CD8^+^ and CD4^+^ T cell subtypes (**Figure 1F**). Expression of PD-1 was higher on CD8^+^ T cells in tumors of GPR55 KO vs. WT mice (**Figure 1G**, left), whereas PD-L1 expression was higher on CD45^-^ cells (**Figure 1H**). GPR55 mRNA expression (which should only derive from GPR55-expressing tumor cells) was significantly lower in tumor samples of GPR55 KO vs. WT mice (**Figure 1I**).

### 3.2 GPR55 deficiency also reduces KPCY55-induced tumor weight and volume

**Figure 2:**
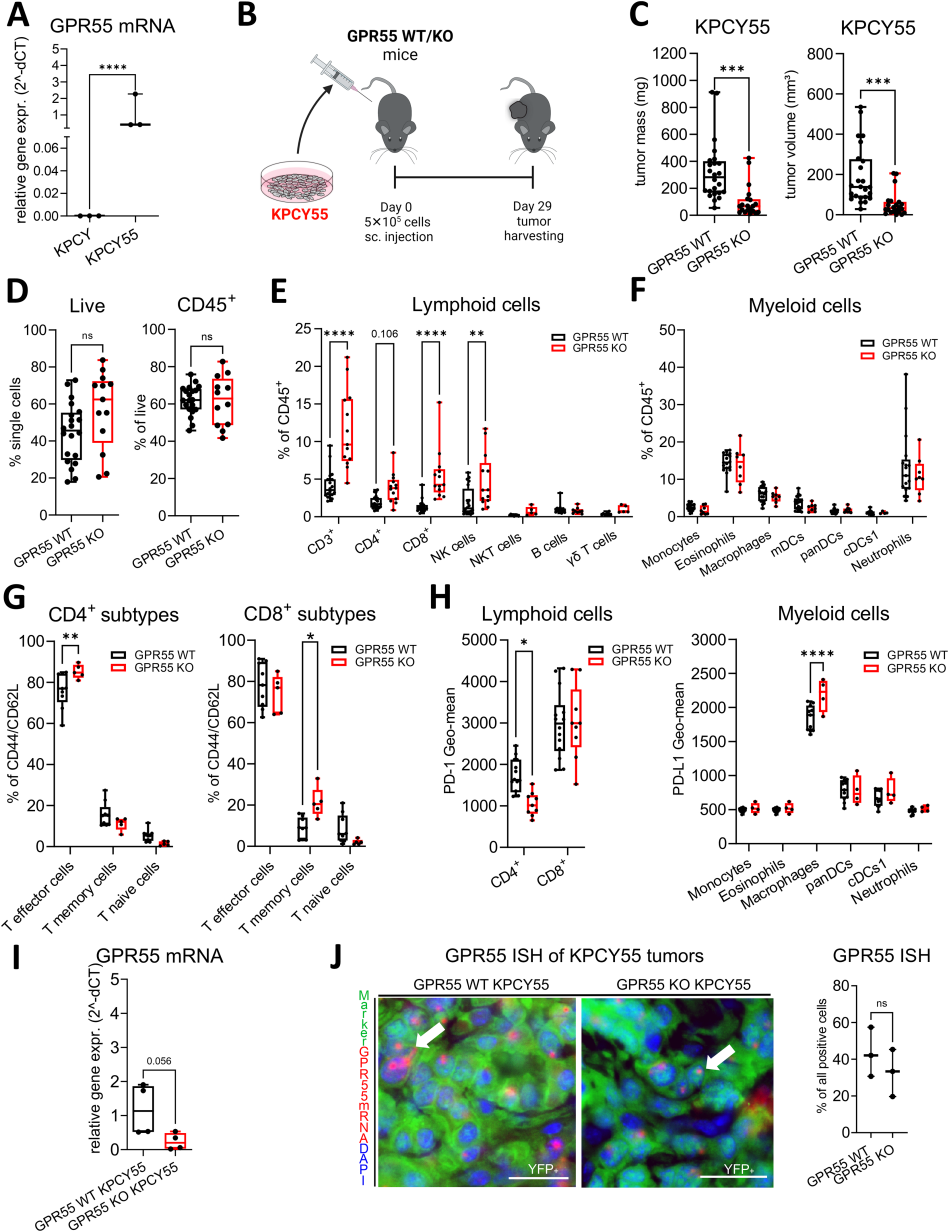
GPR55 deficiency reduces tumor weight and volume in tumors with a GPR55-overexpressing clone (KPCY55). **(A)** Relative gene expression of GPR55 in KPCY and KPCY55 cell lines. n=3. **(B)** GPR55 KO and WT mice were subcutaneously (s.c.) injected on day 0 with 5x10^5^ KPCY55 cells. On day 29, tumors were measured ex vivo and used for analysis. **(C)** Tumor mass and tumor volume from ex vivo measurements of KPCY55 tumors. Data indicate medians, 25^th^ and 75^th^ percentiles, and min-max values from three experiments. n=21-24. **(D–H)** Flow cytometric analysis of single cell suspensions from KPCY55 tumors showing changes in the lymphoid and myeloid cell populations. **(I)** RT-qPCR of KPCY55 whole tumor samples from WT and GPR55 KO mice. n=4. **(J**, left) GPR55 ISH signals (red; representatively indicated by arrows) in tumor cells (YFP tagged) of KPCY55 tumors labeled with anti-GFP antibody (green). Nuclei are stained with DAPI (blue). Calibration bars: 20µm. **(J**, right) Percentage (%) colocalization of GPR55 mRNA ISH signals with KPCY55 tumor cells. Sections from 3 animals were evaluated. Data indicate medians, 25_th_ and 75_th_ percentiles, and min-max values from one **(G)**, two **(H)**, or three experiments **(D–F)**. n=4-24. Statistical differences were assessed by using two-way ANOVA with Šídák’s multiple comparisons test **(E–G)** or unpaired Student’s-t test (**A, C, D, I, J** right). *p<0.05; **p<0.01, ***p<0.001,****p<0.0001; **p<0.01; ****p<0.0001. *WT*, wildtype; *KO*, knockout; CD8+ T mem, CD8+ T memory cells; *panDCs*, pan-dendritic cells; *cDCs1*, dendritic cell type 1; *mDCs*, monocyte-derived dendritic cells; *NK cells*, natural killer cells; ns, not significant *γδ T cells*, gamma delta T cells; *TME*, tumor microenvironment.

Knowing that GPR55 is highly expressed in pancreatic cancer (20, 28), we generated a KPCY cell line (KPCY55) that stably overexpressed GPR55 (**Figure 2A**). KPCY55 cells were injected s.c. into the flanks of mice, and tumors were harvested after 29 days (**Figure 2B**). Similar to KPCY cell-induced tumors, KPCY55 tumors from GPR55 KO mice were smaller in both mass and volume than those from WT mice (**Figure 2C**). Percentages of CD45^+^ cells remained unchanged between the two groups (**Figure 2D**). The number of lymphoid cells was higher in tumors of GPR55 KO mice, with increases in CD3^+^, CD8^+^ T and NK cells (**Figure 2E**). There was no difference between the presence of myeloid cells between the two groups (**Figure 2F**). Among lymphoid subtypes, we measured increases in CD4^+^ T effector and CD8^+^ T memory cells (**Figure 2G**). PD-1 expression on CD8^+^ T cells remained unchanged, but it was lower in CD4^+^ T cells from tumors of GPR55 KO vs. WT mice (**Figure 2H**). On the other hand, expression of PD-L1 was higher on macrophages [known to be the main source of PD-L1 in the TME (33)] in KPCY55 tumors of GPR55 KO mice (**Figure 2H**, right). Total mRNA expression in KPCY55 tumor samples differs widely between GPR55 KO and WT mice, indicating the lack of GPR55 in cells of the TME, and the reduction of tumor cells in KO mice (**Figure 2I**). In **Figure 2J**, representative ISH images and colocalization of GPR55 mRNA with tumor cells are shown in KPCY55 tumors from GPR55 KO and WT mice, demonstrating similar colocalization (**Figure 2J**, right).

### 3.3 GPR55 is present in immune and cancer cells of tumors

**Figure 3:**
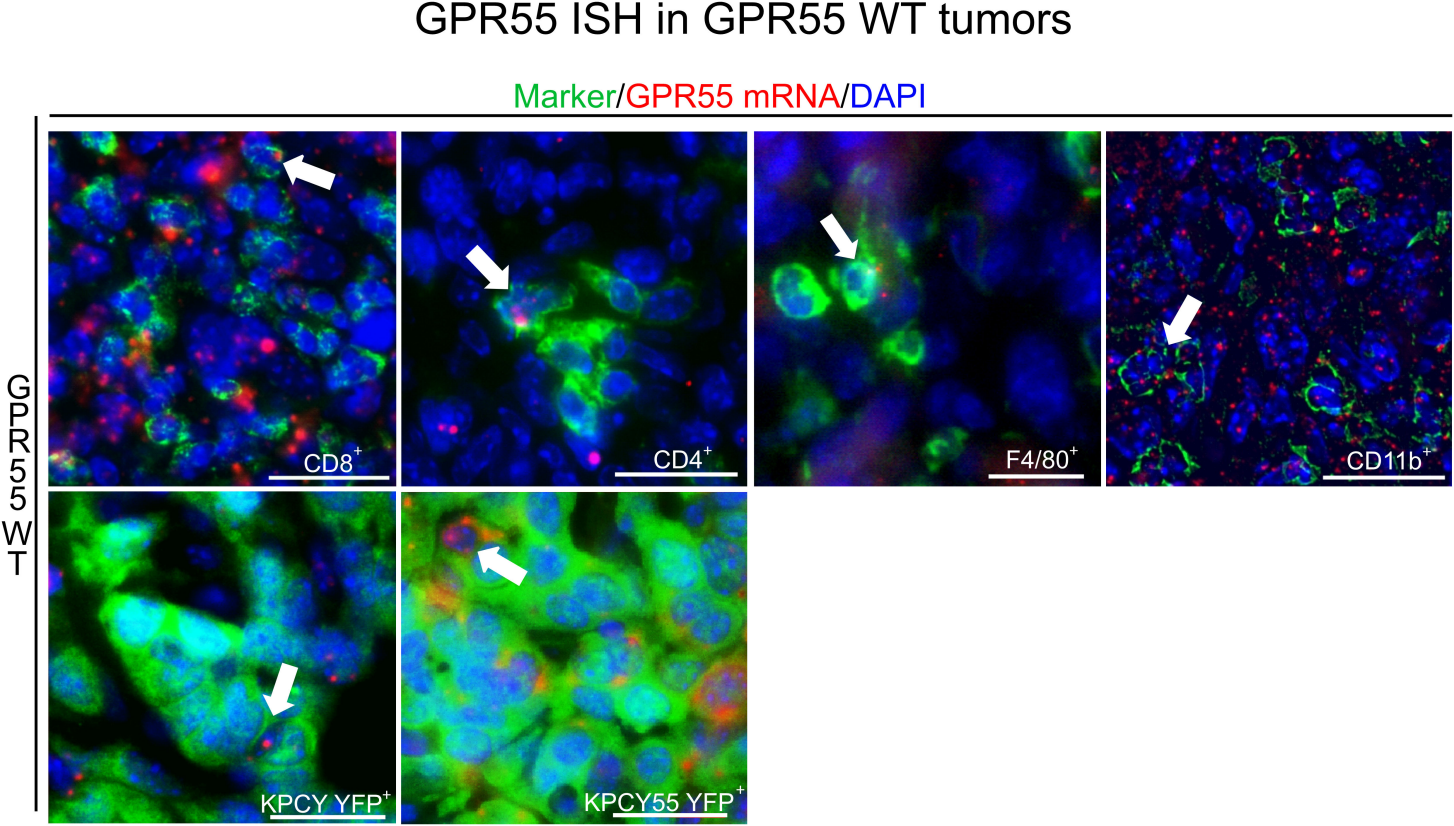
Presence of GPR55 in KPCY and KPCY55 tumors. ISH/immunofluorescence for immune and tumor cells in sections of KPCY and KPCY55 tumors from WT mice. Representative arrows point at cells expressing GPR55 mRNA signals (red). Antibodies against immune cell markers CD8^+^, CD4^+^, CD11b^+^, and F4/80^+^ were used (all in green). Tumor cells (YFP^+^) of KPCY and KPCY55 tumors were labeled with anti-GFP antibody (green). Nuclei were stained with DAPI (blue). Calibration bars=20 µm. 30-900 cells were counted per section; sections from 3 animals used. *ISH*, *In situ* hybridization; *WT*, wildtype; *TME*, tumor microenvironment.

We performed ISH combined with immunofluorescence and localized GPR55 mRNA expression in sections of KPCY and KPCY55 tumors from WT mice (**Figure 3**). GPR55 largely colocalized with CD11b^+^ cells (66.8% ± 7.9%), CD8^+^ T cells (31.6% ± 5.7%), CD4^+^ T cells (43.5% ± 4.9%), but only little with F4/80^+^ macrophages (12.6% ± 4.3%) in KPCY tumors. GPR55 mRNA colocalized with KPCY55 tumor cells by 43.4% ± 13.4%, and with KPCY tumor cells by 31.9% ± 11% (all data are means ± SD).

### 3.4 GPR55 deficiency leads to upregulation of genes involved in immune cell functions in KPCY55-induced tumors

Since changes in the immune TME of GPR55 KO mice were more pronounced in KPCY55 than KPCY tumors, and high expression of GPR55 was reported in human pancreatic cancer (28), we used KPCY55 tumors for RNA-seq evaluation and for further *in vivo* experiments.

**Figure 4:**
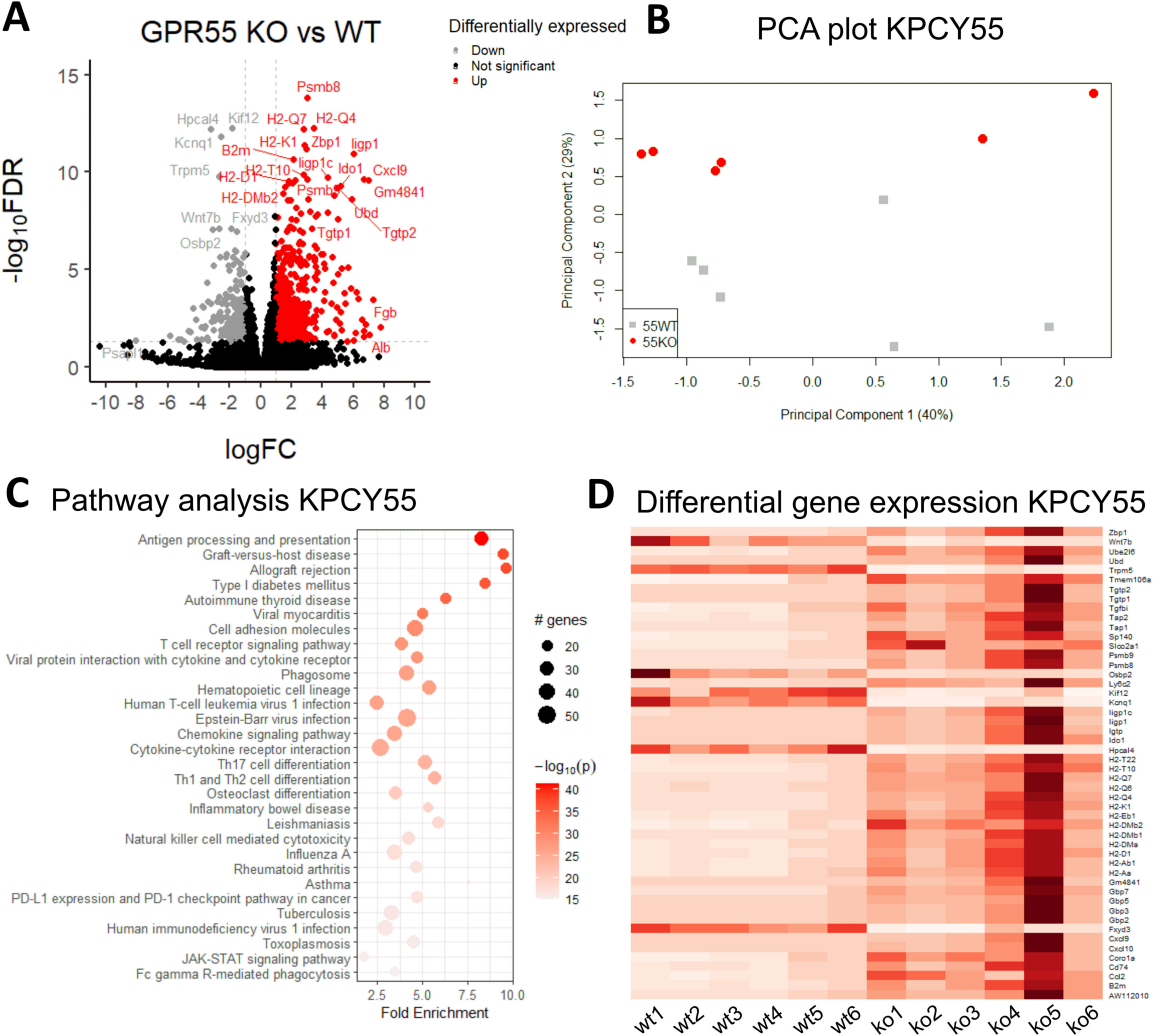
RNA-seq reveals that GPR55 deficiency leads to upregulation of genes involved in enhanced immune cell functions in KPCY55-induced tumors. **(A)** Volcano plot showing the log of fold-change (logFC) on the x axis and the −log_10_ false discovery rate (−log_10_FDR) on the y axis of RNA-seq data. The cut-off values were 1 for fold-change, and 0.05 for FDR, respectively. **(B)** PCA plot of log gene expression data showing the first and second principal components. **(C)** Pathway analysis of RNA-seq data, showing enrichment in processes linked to immunity. The number of genes enriched in a pathway is represented by the size of the dot. The color of the dot represents the −log_10_ (lowest-p) value of the pathway. **(D)** Heatmap of the top 50 differentially expressed genes in KPCY55 tumors from WT and GPR55 KO mice. The resulting Z-score scaling indicates that genes exhibiting high expression relative to the mean have positive Z-scores (dark red), while low-expression genes exhibit negative Z-scores (white). *Ko* (*ko*), knockout; *WT* (*wt)*, wildtype; *TME*, tumor microenvironment.

Gene expression analysis revealed 768 differentially expressed genes (DEGs) in KPCY55 tumors (see volcano plot in **Figure 4A**) of GPR55 KO vs. WT mice, of which 569 were up- and 199 downregulated (|log FC|≥1 and FDR<0.05) (see also **Supplementary Table S3**). PCA analysis of the top 500 genes with greatest significant differences between KPCY55 tumors of GPR55 KO and WT mice revealed clear separation of gene expression profiles (**Figure 4B**). In **Figure 4C** (and **Supplementary Figure S4**), pathway enrichment analysis of RNA-seq data showed that the DEGs particularly correspond, among others, to pathways related to immune cell functions, such as antigen processing/presentation, signaling and differentiation of T cells, NK cell cytotoxicity, and checkpoint protein expression, indicating strong anti-tumor immune responses in KPCY55-induced tumors of GPR55 KO vs. WT mice. Normalized read counts of the top fifty differentially expressed genes from individual mice of each group further highlight the differences in gene expression between the two groups (wt1-wt6 vs. ko1-ko6; **Figure 4D**).

### 3.5 Cell cycle genes and Ki-67 are changed in KPCY55-induced tumors of GPR55 KO vs. WT mice

Our RNA-seq dataset additionally revealed decreased expression of stratifin (Sfn; a protein involved in cell cycle control and used as a biomarker of poor prognosis in PDAC) (34), and cyclin D2 (Ccnd2; involved in cell cycle regulation and malignancy (35)) in KPCY55 tumors of GPR55 KO vs. WT mice (**Supplementary Figure S5A**). Interestingly, there were no changes in the expression of proliferation marker Ki-67 in our bulk RNA-seq data from whole tumor samples (**Supplementary Figure S5A**). However, by measuring Ki-67 mRNA in KPCY55 tumor cells of ISH sections, we observed lower expression in GPR55 KO vs. WT mice (**Supplementary Figure S5B**). GPR55 overexpression did not influence the viability and proliferation of KPCY55 cells in culture (**Supplementary Figures S6A, B**).

### 3.6 Migration of T cells from GPR55 KO mice towards CXCL9 is enhanced

**Figure 5:**
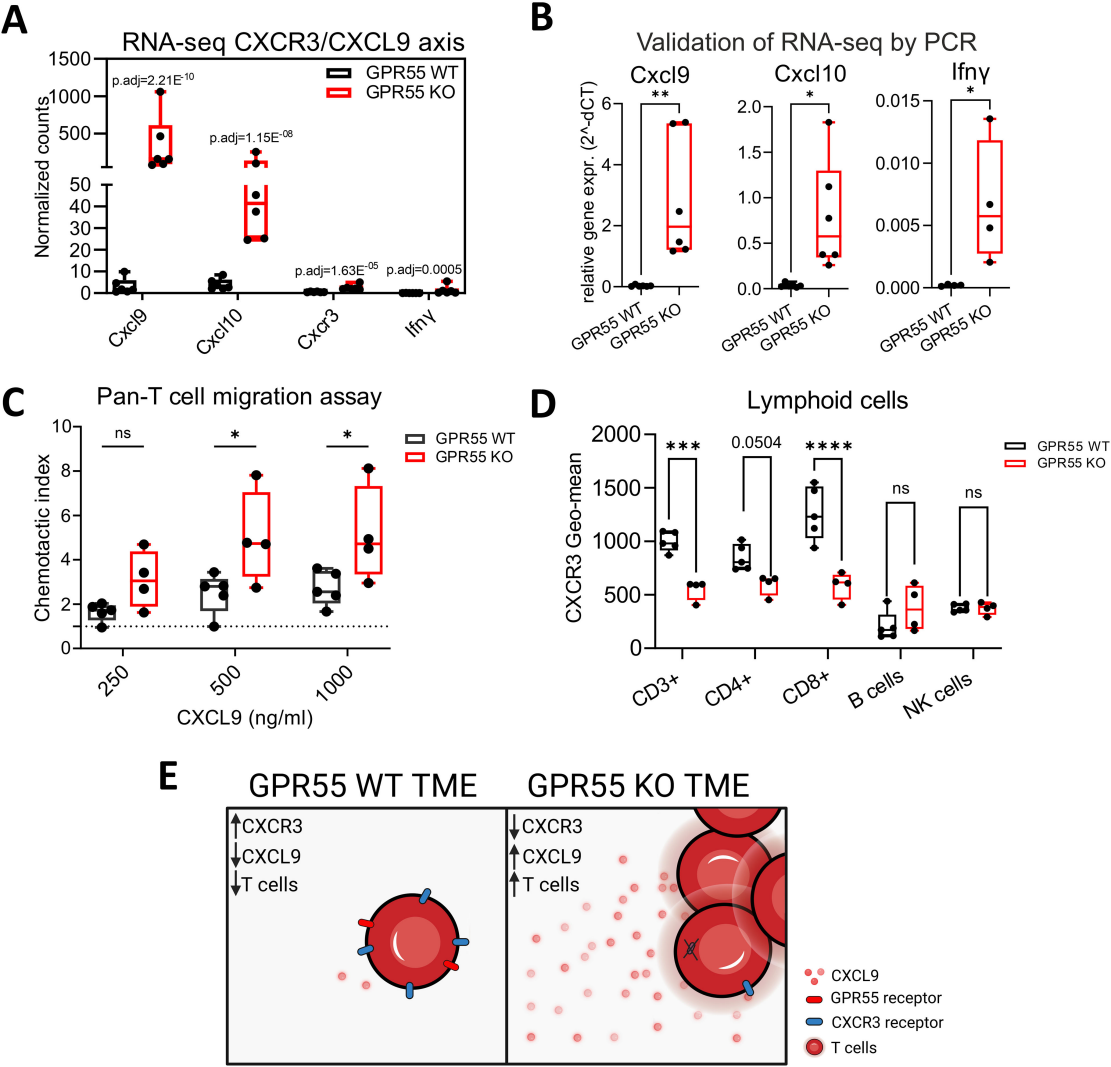
Role of the CXCR3/CXCL9 axis in the migration of T cells. **(A)** Differential expression of Cxcl9, Cxcl10, Cxcr3, and Ifnγ in the TME of KPCY55 tumors from WT and GPR55 KO mice, presented by normalized counts (transcripts per million) with p-adjusted values (p.adj) from bulk RNA-seq. n=6. **(B)** Relative gene expression (2^^-dCT^) of Cxcl9, Cxcl10, and Ifnγ normalized to Hprt. Statistical differences were evaluated by unpaired Student’s t-test, **p<0.01, *p<0.05. n=6. **(C)** Chemotaxis assay of pan-T cells isolated from healthy spleens of GPR55 KO and WT mice, migrating towards different concentrations of CXCL9 in the bottom wells. Statistical differences were evaluated using two-way ANOVA and Tukey’s *post hoc* test. *p<0.05. n=4-5 **(D)** Geometric means of CXCR3 expression on lymphocytes in KPCY55 tumors from WT and GPR55 KO mice. Statistical differences were evaluated using two-way ANOVA with Šídák’s multiple comparisons test. ***p<0.001, ****p<0.0001. n=4-5. In all datasets, data indicate medians, 25^th^ and 75^th^ percentiles, and min-max values. **(E)** Schematic showing the comparison between the GPR55 WT and KO TME. *p.adj*, adjusted p-value; ns, not significant; *WT*, wildtype; *KO*, knockout; *TME*, tumor microenvironment. Created in BioRender. Ristic, **(D)** (2024) https://BioRender.com/j25e259.

Since we observed increased T cell activity in RNA-seq pathway analysis, as well as increased numbers of lymphoid cells in KPCY55 tumors of GPR55 KO mice, we set out to investigate a possible mechanism behind the higher T cell counts, focusing on cytokines/chemokines involved in T cell migration. In our RNA-seq data, significant increases in normalized RNA counts for the T cell-attracting and interferon (IFN)-γ-inducible ligands Cxcl9 and Cxcl10, and for their receptor Cxcr3 (36) were detected (although levels for Cxcr3 and INF-γ were quite low) (**Figure 5A**). We subsequently validated these targets using RT-qPCR (**Figure 5B**) and investigated a potential migratory effect of CXCL9 on T cells involving GPR55. To this end, we assessed the migration of splenic pan-T cells from GPR55 KO and WT mice in response to CXCL9. We counted more T cells from KO than from WT mice migrating towards increasing concentrations of CXCL9 (**Figure 5C**).

Presence of CXCR3 on T cells in KPCY55 tumors was then evaluated using flow cytometry (**Figure 5D**). There was a decrease of CXCR3 on CD3^+^ and CD8^+^ T cells in KPCY55 tumors of GPR55 KO vs. WT mice. Expression of CXCR3 was not different between splenic T cells of healthy WT and GPR55 KO mice (**Supplementary Figure S7**). A schematic summarizing the migration assays is shown in **Figure 5E**.

### 3.7 Deficiency of GPR55 influences anti-PD-1 antibody treatment in mice with KPCY55-induced tumors

**Figure 6:**
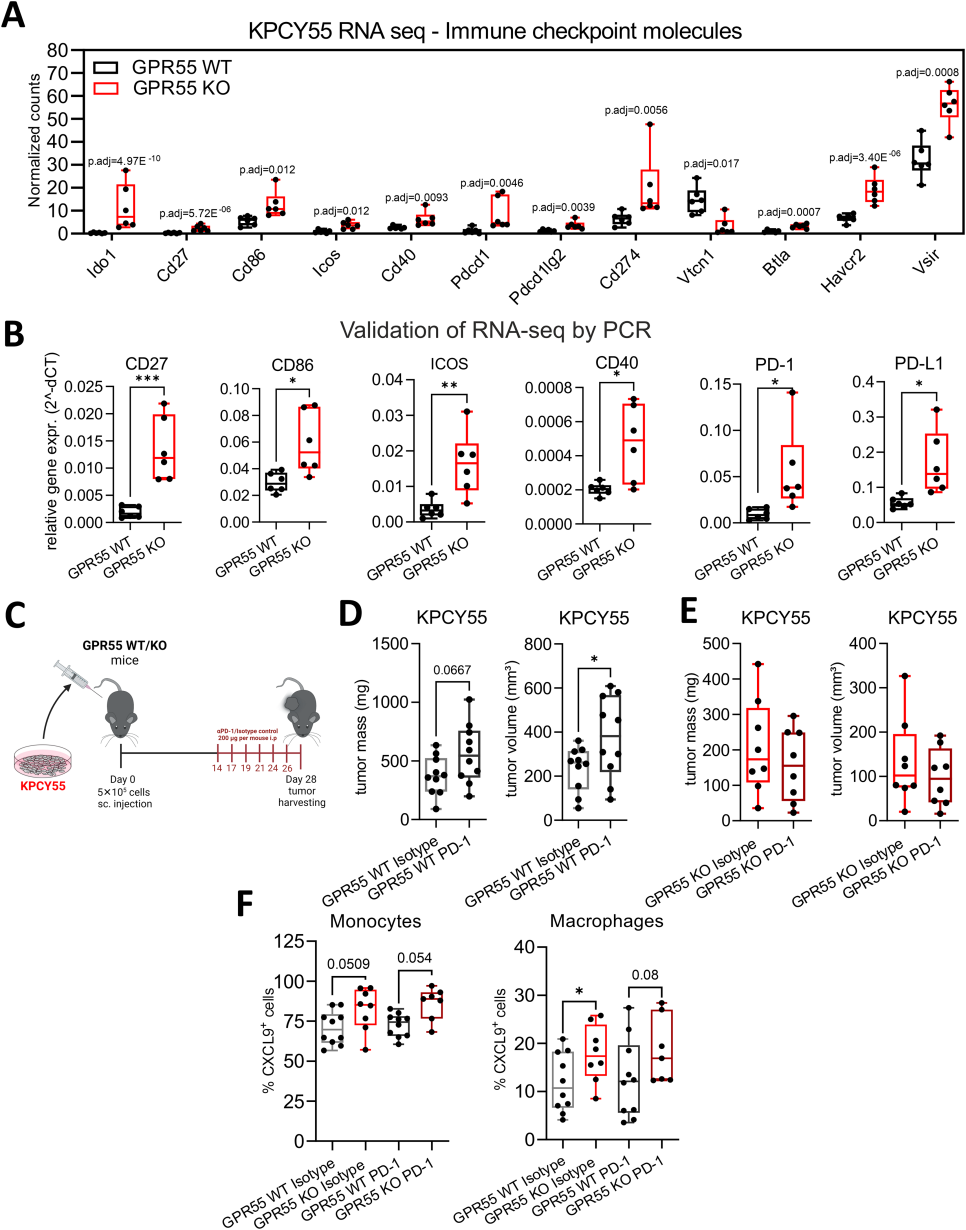
GPR55 deficiency in the TME of KPCY55 tumors influences anti-PD-1 treatment. **(A)** RNA-seq-derived normalized counts (transcripts per million with p-adjusted values (p.adj)) and **(B)** RT-qPCR data (normalized to Hprt) for a select number of immune checkpoint and costimulatory molecules. n=6. **(C)** GPR55 KO and WT mice were subcutaneously (s.c.) injected on day 0 with 5x10^5^ KPCY55 cells. Starting on day 14, six rounds of anti-PD1 antibodies or isotype control were injected i.p. into mice. On day 28, tumors were measured *ex vivo* and used for analysis. **(D, E)** *Ex vivo* measurement of tumor mass and volume in isotype control (Isotype) and anti-PD1 antibody (PD-1) treated mice. n=8-10 per group. Statistical differences were evaluated using unpaired Student’s t-test. **(F)** Flow cytometric analysis of monocytes and macrophages positive for CXCL9 expression in KPCY55 tumors from WT or GPR55KO mice. Statistical differences were evaluated using two-way ANOVA with Šídák’s multiple comparisons test. In all datasets, data indicate medians, 25^th^ and 75^th^ percentiles, and min-max values. *p<0.05, **p<0.01, ***p<0.001. n=7-10. *WT*, wildtype; *KO*, knockout; *p.adj*, adjusted p-value; *PD-1*, anti-PD-1 antibody; *Isotype*, isotype control; *TME*, tumor microenvironment.

In **Figure 2H**, we showed an increase of PD-L1 on macrophages by flow cytometry in KPCY55 tumors of GPR55 KO vs. WT mice. By use of RNA-seq and RT-qPCR, we also detected higher mRNA expression of immune checkpoint and co-stimulatory molecules, e.g., CD27, CD86, ICOS and CD40 (**Figures 6A, B**). Since PD-L1 is one of the most reliable biomarkers for responding to PD-1 blockade (37), we injected KPCY55 cells s.c. into GPR55 KO and WT mice, and after 14 days, treated the mice with 6 rounds of anti-PD-1 antibodies (or isotype control) (**Figure 6C**). We confirmed the results on tumor weight and volume from **Figure 2C** in this set of experiments (see **Supplementary Figures S8A, B**). Notably, when comparing isotype control and anti-PD-1 antibody treatment, we observed that tumor volume and mass increased in WT (**Figure 6D**), but not in GPR55 KO mice (**Figure 6E**). The treatment, however, did not further reduce tumor burden in GPR55 KO mice (**Figure 6E**). To elucidate whether PD-1 inhibition was more effective in GPR55 KO than WT mice, we evaluated the % differences (Δ) in tumor weights/volumes between isotype control and anti-PD-1 antibody treatment in both groups. As a result, we noticed differences in changes of tumor weight/volume that were greater between anti-PD-1-antibody-treated than isotype control-treated mice (p=0.07), suggesting that anti-PD-1 antibodies are more effective in KPCY55 tumors of GPR55 KO than WT mice (**Supplementary Figure S8C**).

With regard to a potential effect of PD-1 blockade on the CXCR3/CXCL9 axis, we found that macrophages from tumors of GPR55 KO mice expressed significantly more CXCL9 than those from tumors of their WT counterparts, while noticing a strong trend in the same direction for monocytes (**Figure 6F**). However, CXCL9 expression did not differ between isotype control and anti-PD-1 antibody treatment (**Figure 6F**) (nor was there a difference in CXCR3 expression on CD3^+^ and CD8^+^ T cells between the groups; see **Supplementary Figure S8D**), indicating that the CXCR3/CXCL9 axis was unaffected by anti-PD1 antibody treatment.

## 4 Discussion

Even though pancreatic cancer is the eleventh most common cancer in the world, it is the seventh leading cause of cancer-related deaths (38). Since involvement of the immune system is low in this type of cancer, new therapeutic approaches are hard to implement, and often do not prolong long-term survival of the patient (39). Recent research suggests that stratification of patients according to immune infiltration could be of benefit (40). As such, a higher number of infiltrating CD8^+^ T cells can predict a better response to immunotherapy (40).

### 4.1 GPR55 in cancer cells

Studies have shown that GPR55 plays a pro-tumorigenic role in many types of cancer (20, 21, 25, 41–43). As to pancreatic cancer, inhibition of GPR55 revealed antitumor effects in a human pancreatic cancer cell line (44). In addition, genetic ablation of GPR55 in a PDAC mouse model significantly prolonged the survival of mice (20). In our experiments, we also saw less Ki-67 expression in tumor cells of GPR55 KO mice, suggesting reduced proliferation through deficiency of GPR55. Its influence on the metabolism of pancreatic cancer cells has been also demonstrated through the actions of (R, R’)-4’-methoxy-1-naphthylfenoterol, which, by modulating GPR55 signaling, altered L-lactose metabolism in PANC-1 cells and mice xenografts (45).

We did not see differences in proliferation and viability between KPCY and GPR55-overexpressing KPCY cells in culture. However, our ISH findings indicated that KPCY tumor cells, although hardly expressing GPR55 mRNA in culture (**Figure 2A**), upregulated GPR55 mRNA *in situ* (**Figure 3**). Importantly, we observed that GPR55 was present in several types of immune cells of the TME, suggesting that GPR55 may influence tumor growth via actions of these cells. In line with this hypothesis, we previously reported smaller tumors and higher infiltration of CD3^+^, CD4^+^, and CD8^+^ T cells, but lower infiltration of myeloid suppressor cells into tumors of GPR55 KO vs. WT mice with colorectal cancer, indicating a possible T cell- suppressive role of GPR55 (18). A pronounced immune response could also be the reason why KPCY55-induced tumors grew slower (appropriate tumor size for harvesting after 29 days) than KPCY-induced tumors in GPR55 KO mice (harvested after 21 days).

### 4.2 GPR55-deficiency favors an anti-tumorigenic immune cell profile

Next to KPCY and KPCY55 tumor cells, we identified GPR55 in numerous immune cells of the TME (CD4^+^ and CD8^+^ T cells, macrophages, and CD11b^+^ myeloid cells) indicating a role for GPR55 in immune cell functions, such as migration and cytokine release (16, 46). Although similar changes in tumor weight and volume were measured for KPCY and KPCY55 tumor-bearing GPR55 KO mice, we observed differences in the composition of their immune TME. Lack of GPR55 in the TME of KPCY55 tumors from KO mice caused a shift in the immune cell profile towards increased numbers of CD8^+^ T and NK cells, which are known for their cytotoxicity against cancer cells (3, 47). Among T cell subtypes, CD4^+^ effector T cells and CD8^+^ memory T cells were increased, indicating enhanced adaptive immune response (48). In KPCY tumors of GPR55 KO mice, we also detected a decrease of neutrophils vs. WT tumors, which fits with the mostly pro-tumorigenic role of neutrophils in pancreatic cancer (49). In addition, KPCY tumors from GPR55 KO mice had a higher number of pan-dendritic cells than tumors from WT mice. Dendritic cells are known to cross-present antigens to CD8^+^ T cells (50), and to enhance T cell priming (51). Spleen from healthy GPR55 KO mice do not show increased levels of CD3^+^ T cells as compared to healthy WT mice (**Supplementary Figure S9**) indicating that the regulation of the immune cell profiles were tumor specific.

### 4.3 RNA-seq data indicate enhanced immune cell functions in KPCY55 tumors of GPR55 KO mice

By employing pathway analysis of differentially-expressed genes, we noticed strong shifts, in particular, towards pathways related to immune cell functions, such as antigen processing/presentation and T cell signaling in KPCY55 tumors of GPR55 KO mice. We further observed upregulation of the immunoproteasome genes Psmb8, 9 and 10, which are known to be involved in enhanced immunogenicity in PDAC (52). These findings may be of importance, as restoring immunity, such as the expression of MHC I on the pancreatic tumor cell surface (53), is of prime interest for new immunotherapies against PDAC. It therefore seems that GPR55 deficiency in the TME favors an anti-tumorigenic environment by overriding T cell suppression.

### 4.4 GPR55 is involved in the migration of T cells towards CXCL9

Although macrophages, neutrophils, mast cells, and tumor cells treated with a GPR55 antagonist decrease their migratory behavior (17, 19, 46, 54), we observed increased migration of pan-T cells, isolated from healthy GPR55 KO mice. This is well in line with a previous study that showed enhanced migration of γδ T cells in GPR55 KO mice, indicating that GPR55 can also function as a migration-inhibitory receptor (16). Our RNA-seq data revealed upregulation of the T cell chemokines Cxcl9/Cxcl10 and their receptor, Cxcr3, in KPCY55 tumors of GPR55 KO mice. CXCL9, CXCL10 and CXCL11 have been previously shown to be effective in supporting an adequate antitumoral response in various cancers (55), although results in pancreatic cancer have been conflicting (56, 57). CXCR3 is almost exclusively expressed in immune cells (36), and together with its ligands, it has been demonstrated to play an important role in anti-tumor T cell development in the spleen (57). Our RNA-seq data showed significantly upregulated Cxcr3 transcripts in KPCY55 tumors of GPR55 KO vs. WT mice, despite the decrease of CXCR3 protein on CD3^+^ and CD8^+^ T cells in the TME. This observation may be explained by a study showing that expression of CXCR3 on T lymphocytes decreases as a consequence of internalization and degradation in the presence of its ligands (58). With regard to the source of CXCL9, we identified monocytes and macrophages as main producers (59), which is in line with a report demonstrating that depletion of CXCL9-expressing tumor-associated macrophages hamper anti-tumor responses (60).

### 4.5 GPR55 influences anti-PD-1 antibody treatment

Pancreatic cancer has one of the lowest response rates to immunotherapy, recently demonstrated in a clinical trial, where 3.1% of patients with metastatic PDAC responded to dual immunotherapy with durvalumab (PD-L1 blocking antibody) and tremelimumab (CTLA-4 blocking antibody), and 0% to durvalumab monotherapy (61). It is known that tumor mutational burden and expression of PD-L1 on tumor cells are robust predictive factors for the success of immunotherapy (37). Our RNA-seq and RT-qPCR data revealed upregulated PD-L1 and PD-1 expression in KPCY55 tumors of GPR55 KO mice. In addition, flow cytometry revealed higher PD-L1 expression on macrophages in KPCY55 tumors from GPR55 KO mice, which suggested a favorable response to immunotherapy. Interestingly, anti-PD-1 antibody treatment increased the tumor burden in WT, but not GPR55 KO mice. The treatment did not further decrease tumor growth in GPR55 KO mice, most likely because it had already been robustly reduced by GPR55 deficiency alone. Regarding the increase in tumor burden in GPR55 WT mice, it should be noted that cancer patients can paradoxically respond with increased tumor progression to immune checkpoint inhibitor therapy (hyperprogressive disease) (62, 63), something that has been now also reported for advanced pancreatic cancer (64). This phenomenon describes an increase in tumor growth post PD-1/PD-L1 blockade, and was observed in several cancers with solid tumors (rev. in Champiat et al.) (62). Reported frequencies lie between 9-29% and are associated with poor outcome (62). Mechanisms for hyperprogressive disease have not been clarified yet but several explanations are discussed, such as T cell exhaustion, a modulation of pro-tumorigenic T cells, or enhanced oncogenic signalling. A case study in advanced pancreatic cancer with hyperprogression treated with PD-1 blockade and chemotherapy identified amplification of MDM4 (a p53 regulating protein) as a risk factor (65). From our experiments, it is not clear what caused hyperprogression. We can only speculate that overexpression of GPR55 in tumor cells may have taken part in the tumor progression of WT mice after PD-1 blockade.

### 4.6 Limitations of the study

One of the limitations of our study is that we only performed mouse experiments but did not use human samples. GPR55, however, has been found to be highly expressed in human PDAC specimens and patient-derived xenografts (20), suggesting that the receptor may also play an important role in human PDAC. In addition, the abundance of CD8^+^ T cells, in particular of the PD-1 CD8^+^ T cell population, in tumors of mouse models injected with KPCY cells, can clearly predict the response to immunotherapy with checkpoint blockers (27). This is in accordance with the situation in human tumors, where a high CD8^+^ T cell population is crucial for a successful immunotherapy with checkpoint inhibitors (47). The findings indicate that our KPCY mouse model can recapitulate the situation of human PDAC, therefore being of high translational value.

## 5 Conclusions

We can demonstrate that deficiency of GPR55 in the TME of murine PDAC tumors leads to improved immune cell infiltration and upregulation of genes involved in T cell activity and function, indicating that TME-derived GPR55 may promote a “cold” tumor. A CXCR3/CXCL9/CXCL10 axis could drive T cell infiltration into KPCY55 tumors of GPR55 KO mice, suggesting that GPR55 suppresses this pathway in PDAC, thereby promoting tumor growth. Tumor cell-derived GPR55 does not seem to play a role in the differences seen in tumor burden between GPR55 KO and WT mice, but we cannot exclude that stromal TME cells like fibroblasts or endothelial cells, which are known to express GPR55, may contribute to it. Finally, our data suggest that GPR55 could be an interesting target for immunotherapies against PDAC by using GPR55 inhibitors as an immune adjuvants to promote T cell trafficking and infiltration, or in an adoptive immunotherapy approach.

## Data Availability

The datasets presented in this study can be found in online repositories. The names of the repository/repositories and accession number(s) can be found below: https://www.ncbi.nlm.nih.gov/geo/, accession number GSE280636.
