# Supplementary material for: GPR55 in the tumor microenvironment of pancreatic cancer controls tumorigenesis
Source: Front Immunol. 2025 Jan 16;15:1513547. doi: 10.3389/fimmu.2024.1513547 (PMC11779727; doi:10.3389/fimmu.2024.1513547)
Supplement: Supplementary file 1 [file DataSheet1.pdf]

Supplementary material

GPR55 in the tumor microenvironment of pancreatic cancer controls tumorigenesis

Dušica Ristić[1], Thomas Bärnthaler[1], Eva Gruden[1], Melanie Kienzl[1], Laura Danner[1], Karolina Herceg[1], Arailym Sarsembayeva[1], Julia Kargl[1], Rudolf Schicho[1]

[1] Division of Pharmacology, Otto Loewi Research Center, Medical University of Graz, Graz, Austria

Table of contents:

Supplementary table S1-3

Supplementary figure S1-9

Supplementary table S1. Mouse antibody panels used in flow cytometry.

Name	Company	Catalog number #	Clone	Volume per test (µl)
**CD103-BV510**	BioLegend	121423	2E7	1.25
**CD11b-BUV737**	BD Biosciences	612801	M1/70	0.625
**CD11c-BV605**	BioLegend	117334	N418	2.50
**CD19-PECy7**	BioLegend	115520	6D5	0.31
**CD206-BV711**	BioLegend	141727	C068C2	0.31
**CD3-BUV395**	BD Biosciences	563565	145-2C11	1.25
**CD44-BUV737**	BD Biosciences	612799	IM7	0.31
**CD45-AF700**	BioLegend	103128	30-F11	0.250
**CD45-BV785**	BioLegend	103149	30-F11	0.313
**CD4-BUV496**	BD Biosciences	564667	GK1.5	0.625
**CD62L-BV605**	BioLegend	104438	MEL-14	1.00
**CD8-PerCPCy5.5**	BioLegend	100734	53-6.7	0.625
**CXCL9-PE**	BioLegend	515604	MIG-2F5.5	2.50
**CXCR3-BV421**	BioLegend	126529	CXCR3-173	1.25
**CXCR3-FITC**	BioLegend	126536	CXCR3-173	1.25
**F4/80-BUV395**	BD Biosciences	565614	T45-2342	1.25
**gdTCR-PECF594**	BD Biosciences	563532	GL3	1.25
**Ly6C-APC**	BioLegend	128015	HK1.4	0.63
**Ly6G-PE/Dazzle594**	BioLegend	127648	1A8	0.60
**MHCII-PerCP-Cy5.5**	BioLegend	107625	M5/114.15.2	0.31
**NKp46-BV510**	BioLegend	137623	29A1.4	2.50
**PD1-APC**	BioLegend	135210	29F.1A12	1.25
**PDL1-PECy7**	BioLegend	124313	10F.9G2	0.63
**SiglecF-BV711**	BD Biosciences	740764	E50-2440	1.25
**SiglecF-PE**	BD Biosciences	562068	E50-2440	1.25

Supplementary table S2. Mouse primers used in RT-qPCR.

Primers	Company	Forward sequence (5'-3')	Reverse sequence (5'-3')
**Mouse Hprt**	Eurofins	TCAGTCAACGGGGGACATAAA	GGGGCTGTACTGCTTAACCAG
**Mouse Gpr55**	Eurofins	CCTCCCATTCAAGATGGTCC	GACGCTTCCGTACATGCTGA
**Mouse Cxcl9**	Eurofins	GGAGTTCGAGGAACCCTAGTG	GGGATTTGTAGTGGATCGTGC
**Mouse Cxcl10**	Eurofins	CCAAGTGCTGCCGTCATTTTC	GGCTCGCAGGGATGATTTCAA
**Mouse Pd-l1**	Eurofins	GCTCCAAAGGACTTGTACGTG	TGATCTGAAGGGCAGCATTTC
**Mouse Pd-1**	Eurofins	ACCCTGGTCATTCACTTGGG	CATTTGCTCCCTCTGACACTG
**Mouse Cd27**	Eurofins	CAGCTTCCCAACTCGACTGTC	GCACCCAGGACGAAGATAAGAA
**Mouse Cd86**	Eurofins	CTGGACTCTACGACTTCACAATG	AGTTGGCGATCACTGACAGTT
**Mouse Cd40**	Eurofins	TGTCATCTGTGAAAAGGTGGTC	ACTGGAGCAGCGGTGTTATG
**Mouse Icos**	Eurofins	TCCAGCAGTTAAAAATGCGATTG	ATCCTCCACTAAGGTTCCTTTCT
**Mouse Ifng**	Eurofins	AGACATCTCCTCCCATCAGCAG	TAGCCAAGACTGTGATTGCGG

Supplementary table S3. Top 100 differentially expressed genes in KPCY55 tumors of GPR55 KO vs. WT mice. In total, gene expression analysis showed 768 differentially expressed genes (DEGs) from bulk RNA-seq, analyzed with the exact test in R Studio 4.4.1. n=6.

Gene_ID	logFC	logCPM	PValue	fdr
**Psmb8**	2.995264	5.30812	4.52E-19	1.49E-14
**Kif12**	-1.8628	4.764125	3.52E-17	5.83E-13
**H2-Q4**	3.449252	6.530203	5.32E-17	5.86E-13
**H2-Q7**	2.754774	3.578076	8.73E-17	6.50E-13
**Hpcal4**	-3.23701	0.728453	9.83E-17	6.50E-13
**Kcnq1**	-2.56388	5.235881	2.86E-16	1.58E-12
**H2-K1**	2.84928	10.11476	8.73E-16	4.12E-12
**Zbp1**	2.930456	4.538435	1.66E-15	6.88E-12
**Iigp1**	6.010788	7.117535	3.20E-15	1.18E-11
**B2m**	2.115912	9.867187	7.07E-15	2.34E-11
**H2-T10**	2.752245	2.718398	4.54E-14	1.36E-10
**Trpm5**	-2.6696	1.025549	6.40E-14	1.76E-10
**Iigp1c**	4.352062	4.22184	7.35E-14	1.87E-10
**Cxcl9**	6.700047	7.409045	1.00E-13	2.21E-10
**Psmb9**	2.997646	5.306798	9.95E-14	2.21E-10
**H2-DMb1**	2.250242	5.865695	1.24E-13	2.57E-10
**Gm4841**	7.001609	4.497769	1.41E-13	2.74E-10
**H2-D1**	1.827541	10.17408	1.56E-13	2.86E-10
**H2-Eb1**	2.029347	8.708615	1.99E-13	3.46E-10
**H2-DMa**	1.788486	5.644675	2.29E-13	3.79E-10
**Ido1**	5.190507	2.60278	3.16E-13	4.97E-10
**H2-DMb2**	1.589396	3.34859	3.84E-13	5.76E-10
**Tgtp2**	5.022571	4.520173	4.20E-13	6.04E-10
**H2-T22**	1.478996	6.655807	8.75E-13	1.21E-09

**Gbp2**	4.783667	7.237889	1.17E-12	1.55E-09
**Tap1**	3.086878	6.326157	2.00E-12	2.54E-09
**Ubd**	5.914522	3.454464	2.09E-12	2.56E-09
**H2-Ab1**	1.915774	8.753128	2.43E-12	2.77E-09
**Ube2l6**	1.76472	4.928696	2.34E-12	2.77E-09
**Slco2a1**	2.299859	6.525225	5.87E-12	6.47E-09
**Igtp**	3.211637	5.770292	1.03E-11	1.10E-08
**Cxcl10**	4.319458	5.426607	1.12E-11	1.15E-08
**Gbp7**	2.585674	5.423167	1.27E-11	1.28E-08
**Gbp5**	3.692437	4.638266	1.58E-11	1.54E-08
**Tmem106a**	0.910517	5.00456	1.89E-11	1.78E-08
**Gbp3**	3.575931	5.44619	1.99E-11	1.83E-08
**Coro1a**	1.117761	6.218186	2.20E-11	1.97E-08
**Cd74**	1.969187	9.799388	2.96E-11	2.51E-08
**Tgtp1**	4.977843	4.432088	2.92E-11	2.51E-08
**AW112010**	2.28396	6.056545	3.63E-11	3.00E-08
**H2-Aa**	1.888672	8.462173	7.47E-11	6.03E-08
**Tap2**	1.90836	4.856642	8.07E-11	6.36E-08
**Fxyd3**	-1.8937	4.692055	1.01E-10	7.44E-08
**Ly6c2**	3.301639	0.923463	1.01E-10	7.44E-08
**Wnt7b**	-2.69263	5.644464	9.92E-11	7.44E-08
**Sp140**	2.103637	2.777309	1.13E-10	8.15E-08
**Ccl2**	1.751626	4.607073	1.28E-10	9.03E-08
**Osbp2**	-3.08175	3.8176	1.33E-10	9.13E-08
**Tgfbi**	0.961899	6.496631	1.35E-10	9.14E-08
**H2-Q6**	2.531759	2.702873	1.45E-10	9.60E-08
**C1ra**	1.715191	4.867681	1.69E-10	1.09E-07
**Tamalin**	-1.54001	6.183515	1.71E-10	1.09E-07
**Klrd1**	2.697767	2.09356	2.04E-10	1.27E-07
**Nod1**	1.344146	4.840371	5.20E-10	3.18E-07
**Ciita**	2.522905	5.036961	6.11E-10	3.67E-07
**Ptpn1**	0.935713	6.326836	7.28E-10	4.30E-07
**Ms4a4b**	2.746746	2.090769	7.77E-10	4.51E-07
**Ifitm3**	1.002208	6.695685	7.96E-10	4.54E-07
**Gimap3**	2.502933	2.837848	8.18E-10	4.58E-07
**Sidt1**	3.49955	-0.70038	1.12E-09	6.16E-07
**Lat**	1.893301	2.163919	1.36E-09	7.36E-07
**Il2rg**	1.517641	4.513233	1.42E-09	7.58E-07
**Sp110**	1.726616	3.751481	1.47E-09	7.74E-07
**Nlrc5**	3.640198	4.504789	1.60E-09	8.28E-07
**Kcne3**	-1.64216	4.904595	2.09E-09	1.06E-06
**Grap2**	2.507565	2.091783	2.31E-09	1.15E-06
**Onecut3**	-1.61498	6.491526	2.33E-09	1.15E-06
**Clec12a**	2.125708	2.970034	2.42E-09	1.16E-06
**Il21r**	1.427276	3.108438	2.41E-09	1.16E-06
**Selplg**	1.25247	4.751425	3.04E-09	1.43E-06
**Cyth4**	1.075966	5.930888	3.25E-09	1.49E-06
**Patj**	-1.02166	5.469034	3.22E-09	1.49E-06
**Gbp4**	4.032467	5.599846	3.44E-09	1.56E-06
**Pkp1**	-2.19746	3.230394	3.60E-09	1.61E-06
**Cdh13**	-0.96069	6.408217	3.69E-09	1.63E-06
**Gm9574**	3.931692	-0.21078	3.74E-09	1.63E-06
**H2-M3**	1.6719	4.195139	4.21E-09	1.81E-06
**Ifi47**	1.897292	5.29904	4.42E-09	1.87E-06
**Slc38a1**	1.971656	3.665383	4.63E-09	1.94E-06
**H2-Q1**	3.109154	0.59787	4.90E-09	2.03E-06
**Clu**	-1.14079	12.7723	5.29E-09	2.16E-06
**Gm13648**	-1.7814	3.029204	5.35E-09	2.16E-06
**Irgm2**	2.206872	5.544335	5.48E-09	2.18E-06
**Arhgap9**	1.121183	4.032809	5.77E-09	2.20E-06
**C1s1**	1.913224	5.36638	5.69E-09	2.20E-06
**Cd3g**	3.129894	2.549363	5.87E-09	2.20E-06
**F5**	-2.60272	3.764912	5.83E-09	2.20E-06
**Slc4a11**	-2.26596	5.342239	5.87E-09	2.20E-06
**Serpina3f**	4.630093	0.747673	6.01E-09	2.23E-06

**H2-Eb2**	3.410936	-0.48034	6.22E-09	2.29E-06
**H2-Q5**	2.508617	1.772034	6.57E-09	2.39E-06
**Snx20**	1.170065	3.699837	6.81E-09	2.45E-06
**Lcp1**	0.980001	6.939219	7.11E-09	2.53E-06
**Rasal3**	1.62967	2.837777	7.23E-09	2.54E-06
**Slco3a1**	3.492298	5.610417	7.59E-09	2.64E-06
**Stat1**	1.972441	5.532439	8.27E-09	2.85E-06
**Hap1**	1.818798	2.441918	9.12E-09	3.08E-06
**Spn**	2.04751	4.160045	9.12E-09	3.08E-06
**Havcr2**	1.495698	3.696018	1.02E-08	3.40E-06

Supplementary figure S1

Lymphoid cell gating in KPCY and KPCY55 mouse tumor samples.

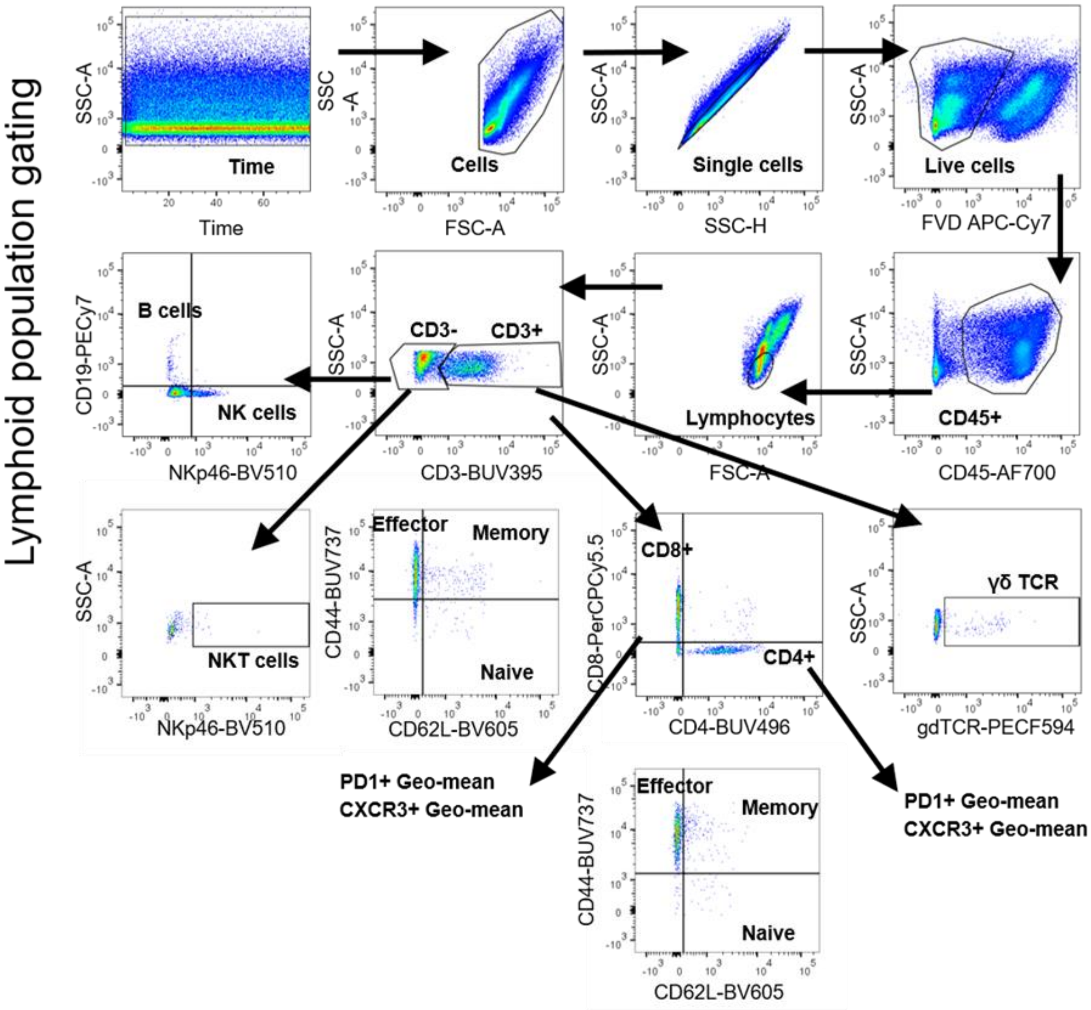

Only CD45^{+} live cells were included in analysis. The populations were defined as:

CD3^{+} T cells - CD3^{+}/Lymphocytes

CD3^{+} CXCR3^{+} T cells - CXCR3 Geo-mean of CD3^{+} T cells

CD4⁺ T cells - CD4⁺/CD3⁺/ Lymphocytes

CD4⁺ CXCR3⁺ T cells – CXCR3 Geo-mean of CD4⁺ T cells

CD4⁺ PD-1⁺ T cells – PD-1 Geo-mean of CD4⁺ T cells

CD4⁺ Effector T cells - CD44⁺CD62L⁻/CD4⁺/CD3⁺/ Lymphocytes

CD4⁺ Memory T cells - CD44⁺CD62L⁺/CD4⁺/CD3⁺/ Lymphocytes

CD4⁺ Naive T cells - CD44⁻CD62L⁺/CD4⁺/CD3⁺/ Lymphocytes

CD8⁺ T cells – CD8⁺/CD3⁺/ Lymphocytes

CD8⁺ Effector T cells - CD44⁺CD62L⁻/CD8⁺/CD3⁺/ Lymphocytes

CD8+ Memory T cells - CD44⁺CD62L⁺/CD8⁺/CD3⁺/ Lymphocytes

CD8+ Naive T cells - CD44⁻CD62L⁺/CD8⁺/CD3⁺/ Lymphocytes

CD8⁺ CXCR3⁺ T cells – CXCR3 Geo-mean of CD8⁺ T cells

CD8⁺ PD-1⁺ T cells – PD-1 Geo-mean of CD8⁺ T cells

γδ T cells – gdTCR⁺/CD3⁺

CD3⁻ cells – CD3⁻/ Lymphocytes

NK cells – NKp46⁺CD19⁻/CD3⁻/Lymphocytes

B cells – NKp46⁻CD19⁺/ CD3⁻/Lymphocytes

NKT cells - NKp46⁻/CD3⁻/Lymphocytes

Supplementary figure S2

Myeloid and tumor cell gating in the KPCY and KPCY55 mouse tumor samples.

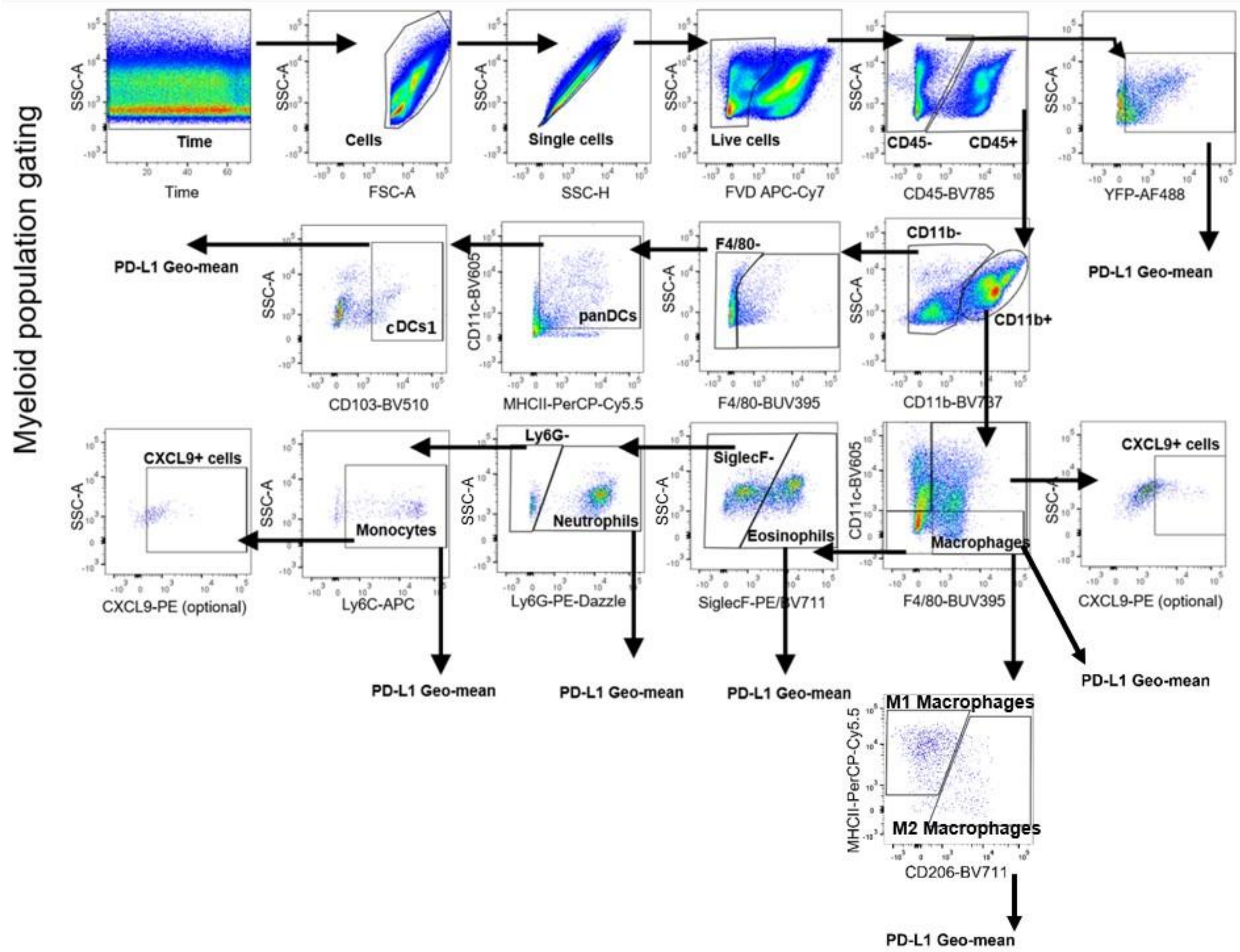

Only live cells were included in analysis. The populations were defined as:

KPCY/KPCY55 tumor cells – YFP$^+$/CD45$^-$

KPCY/KPCY55 PD-L1$^+$ tumor cells – PD-L1 Geo-mean of KPCY/KPCY55 cells

Pan-DCs (pan dendritic cells) – MHCII$^+$ CD11c$^+$/ F4.80$^-$/CD11b$^-$/CD45$^+$

Pan-dendritic PD-L1$^+$ cells - PD-L1 Geo-mean of Pan-DCs

cDCs1 – CD103$^+$/MHCII$^+$ CD11c$^+$/ F4.80$^-$/CD11b$^-$/CD45$^+$

Dendritic PD-L1$^+$ cell type 1 – PD-L1 Geo-mean of cDCs1

Macrophages – F4.80$^+$ CD11c$^+$/CD11b$^+$/CD45$^+$

M1 macrophages $^-$ CD206$^-$/ F4.80$^+$ CD11c$^+$/CD11b$^+$/CD45$^+$

M2 macrophages - CD206$^+$/ F4.80$^+$ CD11c$^+$/CD11b$^+$/CD45$^+$

PD-L1$^+$ macrophages - PD-L1 Geo-mean of macrophages

CXCL9$^+$ macrophages – CXCL9$^+$/F4.80$^+$ CD11c$^+$/CD11b$^+$/CD45$^+$

Eosinophils – SiglecF$^+$/CD11c$^-$/CD11b$^+$/CD45$^+$

PD-L1$^+$ eosinophils - PD-L1 Geo-mean of eosinophils

Neutrophils – Ly6G$^+$/SiglecF$^-$/CD11c$^-$/CD11b$^+$/CD45$^+$

PD-L1$^+$ neutrophils - PD-L1 Geo-mean of neutrophils

Monocytes – $Ly6C^+/Ly6G^-/SiglecF^-/CD11c^-/CD11b^+/CD45^+$

PD-L1⁺ monocytes - PD-L1 Geo-mean of monocytes

CXCL9⁺ monocytes – $CXCL9^+/Ly6C^+/Ly6G^-/SiglecF^-/CD11c^-/CD11b^+/CD45^+$

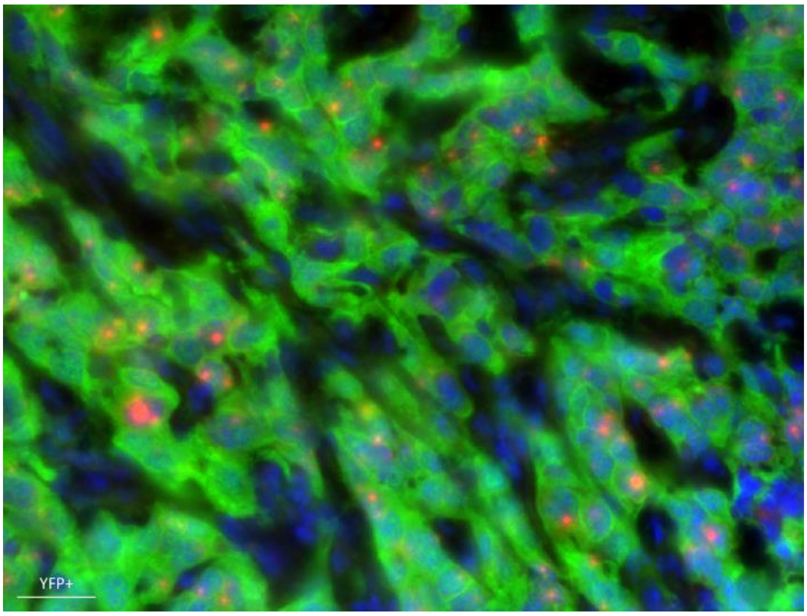

Supplementary figure S3

GPR55 mRNA expression in tumors of GPR55 KO mice is limited to tumor cells. In situ hybridization (ISH)/immunofluorescence of tumor (YFP^+) cells in sections of KPCY55 tumors from GPR55 KO mice Scale=20 μm. Red - GPR55 mRNA, green - YFP^+ tumor cells stained with anti-GFP antibody, blue – nuclear DAPI.

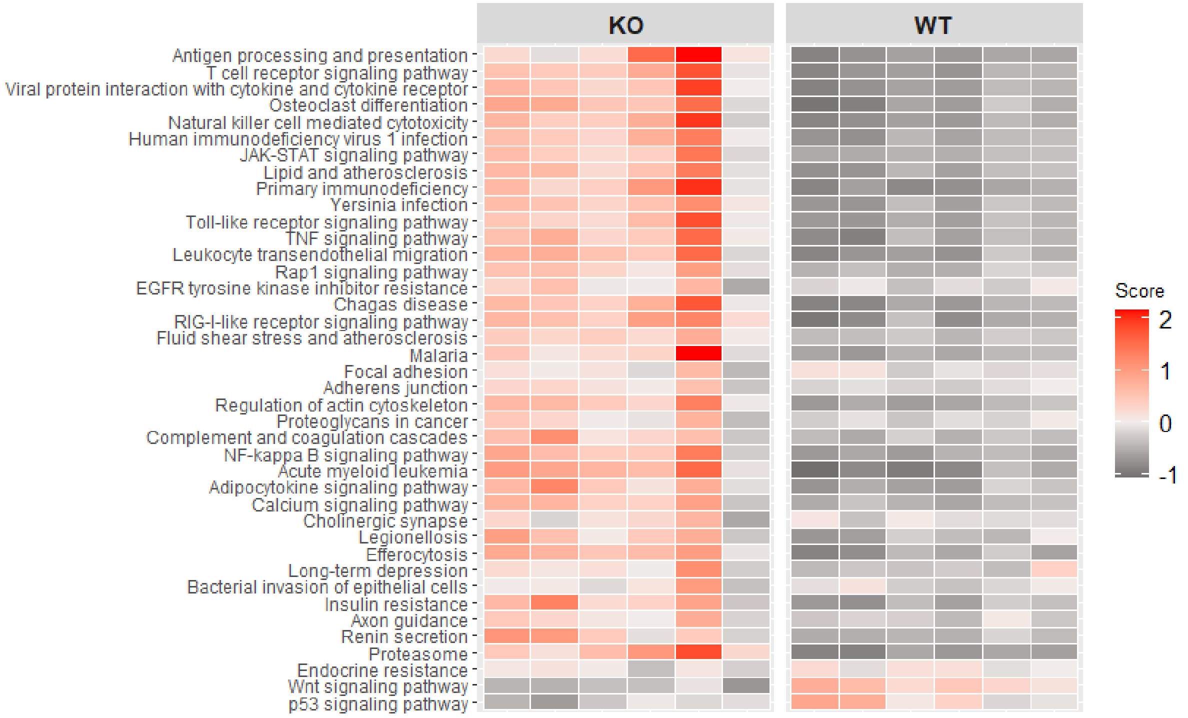

Supplementary figure S4

Top 40 enriched pathways of bulk RNA-seq data assessed in KPCY55 tumors from individual GPR55 KO vs. WT mice. Pathway analysis was done in RStudio 4.4.1 and pathfindR (version 2.4.1). n=6/group.

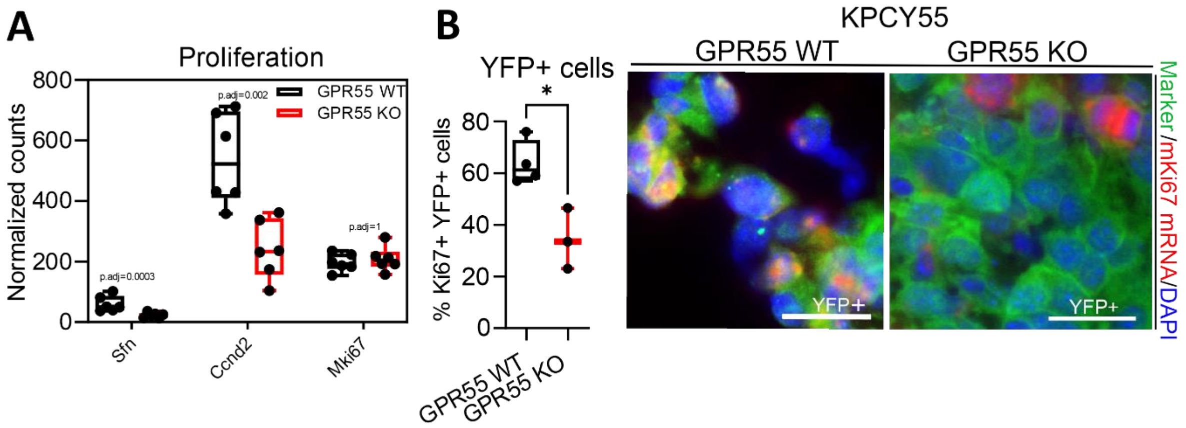

Supplementary figure S5

The expression of cell cycle genes and proliferation marker Ki-67 in KPCY55 tumors of GPR55 WT and KO mice.

(A) Differential expression of stratifin (Sfn), cyclin D2 (Ccnd2), and Ki-67 (Mki67) in KPCY55 tumors of GPR55 KO and WT mice, presented by normalized counts from bulk RNA-seq (p-adjusted values [p.adj] from exact test). n=6. **(B)** Quantification of colocalization of Ki-67 mRNA with tumor YFP^{+} cells, and representative ISH/immunofluorescence images of KPCY55 tumor sections from WT and GPR55 KO mice. Red - GPR55 mRNA, green - YFP+ tumor cells stained with anti-GFP antibody, blue – nuclear DAPI. Scale=20 µm. Data indicate medians, 25th and 75th percentiles, and min-max values. n=3-4 animals. Statistical differences were evaluated by using unpaired Student's t-test, *p<0.05.

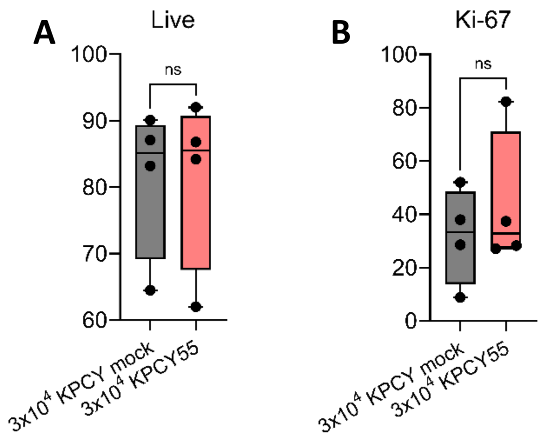

Supplementary figure S6

GPR55 overexpression does not influence the viability and proliferation of KPCY55 cells in culture. 3 x 10^4 KPCY55 and KPCY (+ mock vector) cells were seeded on day 0. After 48 hrs, cells were collected for flow cytometric analysis. (**A**) Percentage of FVD^+ live cells (*FVD*, Fixable Viability Dye eFluor™ 780. (**B**) Percentage of Ki-67 in KPCY55 and KPCY mock cells. Data indicate medians, 25th and 75th percentiles, and min-max values. Statistical differences were evaluated by using unpaired Student's t-test. n=4.

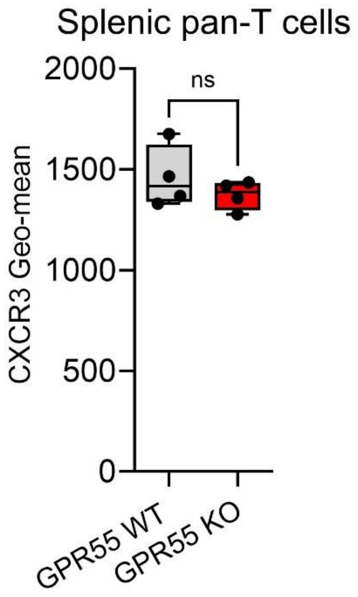

Supplementary figure S7

CXCR3 expression in pan-T cells from healthy spleen. Geometric mean of CXCR3 expression on splenic pan-T lymphocytes from GPR55 WT and KO mice, analyzed by flow cytometry. Data indicate medians, 25th and 75th percentiles, and min-max values. Statistical differences were evaluated by using unpaired Student's t-test. n=4.

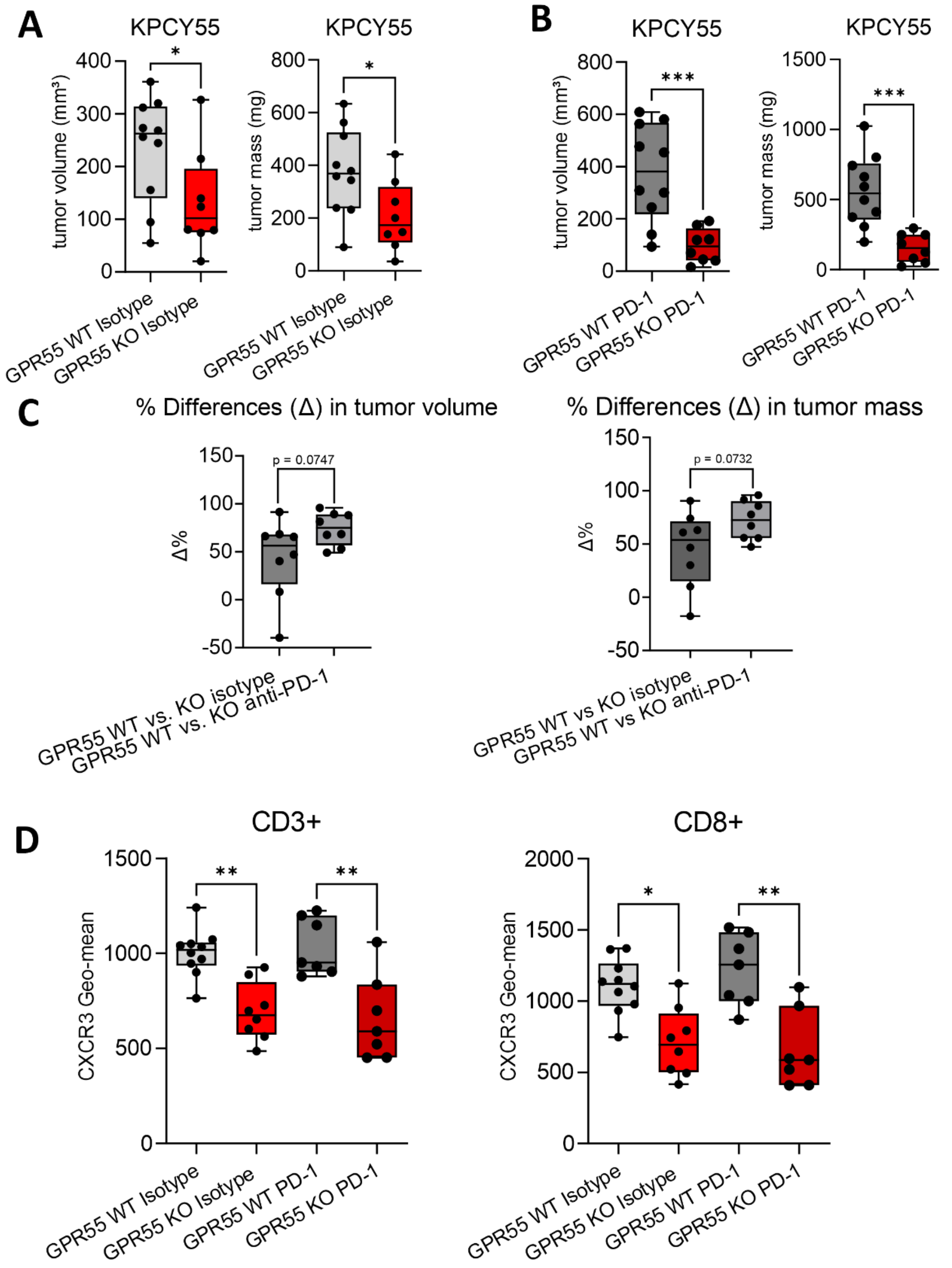

Supplementary figure S8

GPR55 deficiency in the TME impacts anti-PD-1 treatment. *Ex vivo* measurement of (**A**) tumor volume and mass of isotype control (Isotype)- and (**B**) anti-PD1 antibody (PD-1)-treated mice. n=7-10 per group. Statistical differences were evaluated using unpaired Student's t-test. *p<0.05; ***p<0.0001. (**C**) Comparison of % differences (Δ) in tumor volume and mass between GPR55 WT vs. KO isotype control treatment (isotype) and GPR55 WT vs. KO anti-PD-1 antibody (anti-PD-1) treatment (p=0.07; Student's t-test). n=7-10. (**D**) Flow cytometric analysis of CXCR3 on immune cells from KPCY55 tumors. n=7-10 per group. Statistical differences were evaluated by using two-way ANOVA with Šídák's multiple comparisons test, *p<0.05; **p<0.01. In all figures, data indicate medians, 25th and 75th percentiles, and min-max values.

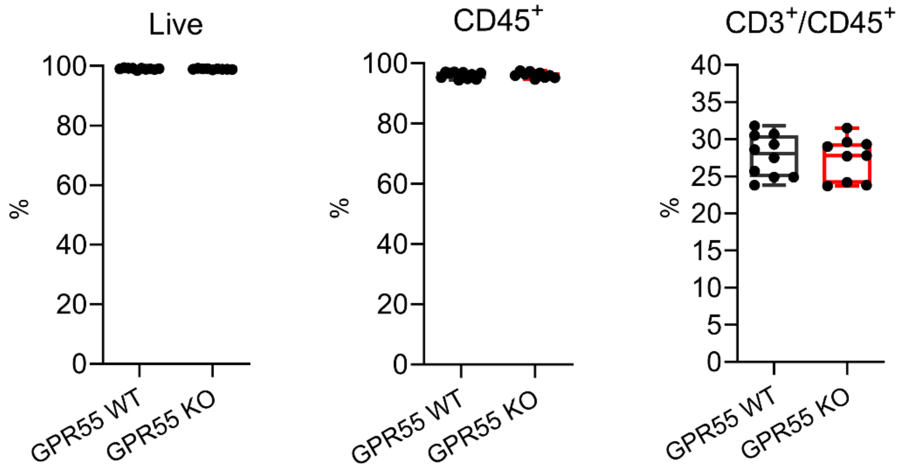

Supplementary figure S9. Flow cytometric analysis of GPR55 WT/KO spleen in steady state. Percentage of Live, $CD45^+$ and $CD3^+$ of $CD45^+$ in the spleens of GPR55 WT or KO mice. Data indicate medians, 25th and 75th percentiles, and min-max values.

Graphical abstract

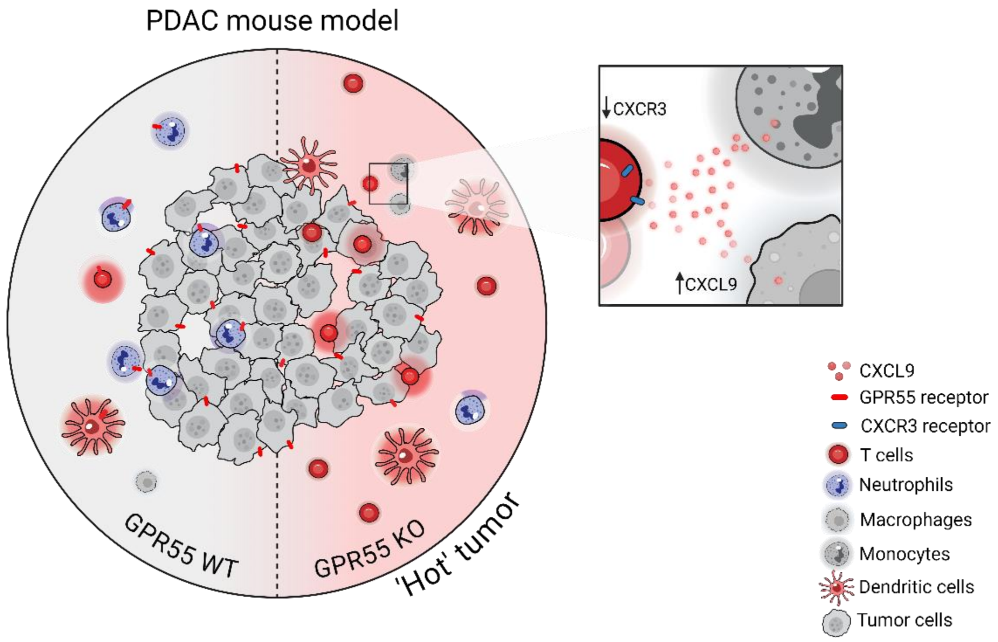

Legend

GPR55 expression in the tumor microenvironment (TME) promotes tumor growth in experimental PDAC by inhibiting T cell infiltration and activity, leading to a pro-tumorigenic environment. GPR55 deficiency in the TME of GPR55 KO mice enhances T cell infiltration and anti-tumor immunity. A CXCR3/CXCL9 axis could drive T cell infiltration into tumors of GPR55 KO mice. Created in BioRender. Ristic, D. (2024) BioRender.com/y17t385.

Authors

Dušica Ristić, Thomas Bärnthaler, Eva Gruden, Melanie Kienzl, Laura Danner, Karolina Herceg, Arailym Sarsembayeva, Julia Kargl, Rudolf Schicho

Correspondence

rudolf.schicho@medunigraz.at
